# Integrated metabolomics and metagenomics analysis of plasma and urine identified microbial metabolites associated with coronary heart disease

**DOI:** 10.1038/srep22525

**Published:** 2016-03-02

**Authors:** Qiang Feng, Zhipeng Liu, Shilong Zhong, Ruijun Li, Huihua Xia, Zhuye Jie, Bo Wen, Xiaomin Chen, Wei Yan, Yanqun Fan, Zhenyu Guo, Nan Meng, Jiyan Chen, Xiyong Yu, Zhiwei Zhang, Karsten Kristiansen, Jun Wang, Xun Xu, Kunlun He, Guanglei Li

**Affiliations:** 1 BGI-Shenzhen, Shenzhen, 518083, China; 2 Department of Oral Microbiome, School of Stomatology, Shandong University, Shandong Provincial Key Laboratory of Oral Tissue Regeneration, Jinan, China; 3 Shenzhen Engineering Laboratory of Detection and Intervention of human intestinal microbiome; 4 College of Life Sciences, University of Chinese Academy of Sciences, 19A Yuquan Road, Shijingshan District, Beijing, 100094, China; 5 Medical Research Center of Guangdong General Hospital, Guangzhou, Guangdong, China; 6 Guangdong Cardiovascular Institute, Guangdong Academy of Medical Sciences, Guangzhou, Guangdong, China; 7 Department of cardiology, Chinese PLA general hospital, Fuxing Road 28, Beijing, 100853, China; 8 Shenzhen Key Laboratory of Human Commensal Microorganisms and Health Research, BGI-Shenzhen, Shenzhen, China; 9 Department of Biology, University of Copenhagen, Ole MaaløesVej 5, 2200 Copenhagen, Denmark; 10 Princess Al Jawhara Center of Excellence in the Research of Hereditary Disorders, King Abdulaziz University, Jeddah 21589, Saudi Arabia; 11 Macau University of Science and Technology, Avenida Wai long, Taipa, Macau 999078, China; 12 Department of Medicine, University of Hong Kong, Hong Kong

## Abstract

Coronary heart disease (CHD) is top risk factor for health in modern society, causing high mortality rate each year. However, there is no reliable way for early diagnosis and prevention of CHD so far. So study the mechanism of CHD and development of novel biomarkers is urgently needed. In this study, metabolomics and metagenomics technology are applied to discover new biomarkers from plasma and urine of 59 CHD patients and 43 healthy controls and trace their origin. We identify GlcNAc-6-P which has good diagnostic capability and can be used as potential biomarkers for CHD, together with mannitol and 15 plasma cholines. These identified metabolites show significant correlations with clinical biochemical indexes. Meanwhile, GlcNAc-6-P and mannitol are potential metabolites originated from intestinal microbiota. Association analysis on species and function levels between intestinal microbes and metabolites suggest a close correlation between *Clostridium sp. HGF2* and GlcNAc-6-P, *Clostridium sp. HGF2*, *Streptococcus sp. M143*, *Streptococcus sp. M334* and mannitol. These suggest the metabolic abnormality is significant and gut microbiota dysbiosis happens in CHD patients.

Coronary heart disease (CHD) is the top risk factor in modern society with annual mortality rate overpassing the sum of all types of cancers. The majority of cardiovascular deaths occurrence are related to the extent of people’s awareness of their own medical conditions and are due to lack of in-time treatment as demonstrated by a five-year follow-up study by MaGiCAD cohort^1^. The challenge for early diagnosis and prevention of CHD lies in the fact that there are no reliable non-invasive biomarkers. The “gold standard” for diagnosis of CHD is still coronary angiography which is invasive and accompanied by many deadly side effects^2^,^3^. This limited the large population screening and the CHD risk prediction at early stage. So non-invasive and highly accurate approaches to diagnose and predict CHD are urgently needed.

Previous research has reported that fatty acids play important roles in the metabolism process of heart; they are predominant substrates, accounting for 60–90% cardiac ATP synthesis, for cardiac ATP generation by mitochondrial oxidative phosphorylation under normal physiological conditions^4^. Cardiovascular diseases (CVD) like coronary heart disease and cardiac failure undergo a “metabolic shift” as a consequence of both intrinsic and extrinsic perturbations. Increased low-density lipoprotein cholesterol (LDL-C) has previously been considered as one of the major risk factors for CHD^5^. The fact that core defects in cardiovascular disease are lipids metabolism^6^ makes metabolomics a particularly promising method to study these types of diseases.

Metabolomics is an innovative and high-throughput bioanalytical method aiming to identify and quantify small molecules (molecular weight less than 1500 Daltons) present in any biological system or any specific physiological state. Two major analytical techniques, nuclear magnetic resonance (NMR) and mass spectrometry (MS), have been widely used in endogenous compounds measurement at an exponential increasing rate in last decade^7^. MS-based techniques have made rapid progress and have been used more frequently compared with NMR since 2005 because of the following advantages: higher sensitivity, more coverage of the metabolome, improved metabolites identification and discrimination capacity, and modularity to perform compound-class-specific analysis^8^. MS is mostly used in conjunction with chromatography, such as gas chromatography mass spectrometry (GC–MS) and liquid chromatography mass spectrometry (LC–MS).

Recent studies in CVD suggest that there are direct links between diet, the gut microbiome and biological events associated with CVD. Choline and phosphatidylcholine from diet could be metabolized to trimethylamine (TMA) by intestinal microbiota which would be further metabolized to a proatherogenic factor – trimethylamine-N-oxide (TMAO), which has proved to accelerate atherosclerosis in mice by chronic dietary L-carnitine and associate with increased risks for both prevalent CVD and incident major adverse cardiac events (myocardial infarction, stroke or death)^9^,^10^.

To explore potential characteristic metabolites signatures associated with CHD, non-targeted metabolomics technique is performed to discover potential metabolites by analysis of plasma and urine samples, and metagenomics technology is applied to further validate the potential metabolites originated from the fecal metagenomics data of CHD patients and healthy subjects. The workflow is shown in Fig. 1. Statistical and bioinformatics methods are used to identify significantly different metabolites that can discriminate CHD cases from healthy controls. Hierarchical cluster analysis (HCA) is performed to identify metabolites clusters contributing to phenotype separation and spearman correlation analysis is applied to identify potential biomarkers’ correlations related to abnormal functions. The identified significantly changed metabolites are validated using purchased standards. Several significantly differential expressed metabolites are correlated with intestine flora on ECs, KOs and species levels. This study demonstrates the strong power of metabolomics in potential noninvasive biomarkers discovery from biofluids of patients. Integrated analysis of metabolomics and metagenomics could pave a new way to reveal the interactions between host and gut microbiobes.

## Results

### Metabolic profiles of plasma and urine samples

Untargeted metabolomics analysis was performed for the plasma and urine samples from 59 CHD patients and 43 healthy controls. The participants’ clinical information was listed in Supplementary Table S1. Albumin (ALB, *p.value* = *4.06E-05*), alanine aminotransferase (ALT, *p.value* = *0.02*), total protein (TP, *p.value* = *1.10E-14*), low-density lipoprotein (LDLC, *p.value* = *0.01*), cholesterol (CHOL, *p.value* = *4.69E-05*), high-density lipoprotein (HDLC, *p.value* = *1.25E-07*), apolipoprotein b (APOB, *p.value* = *1.17E-03*) and apolipoprotein a (APOA, *p.value* = *0.01*) were found to be significantly different in CHD patients from healthy controls by two-tailed student t-test.

**Figure 1:**
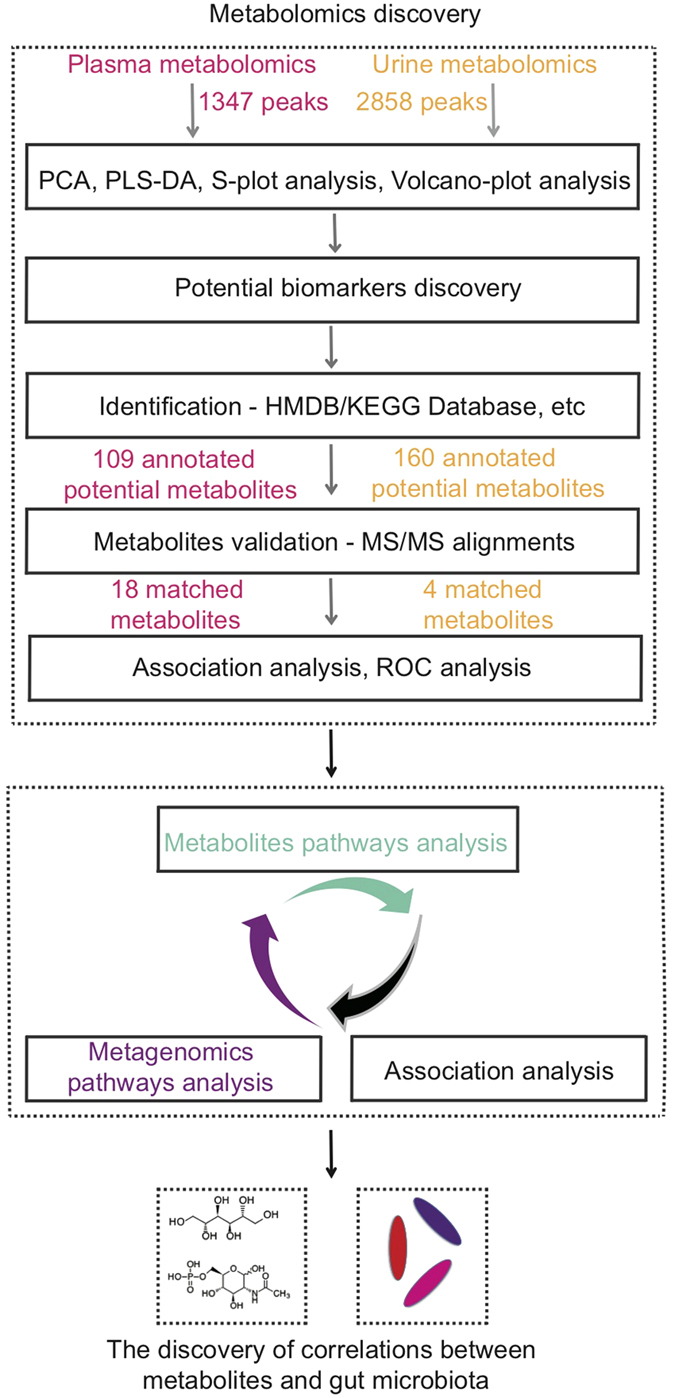
Overview of the study. Non-targeted metabolomics technique is performed to discover potential metabolites in plasma and urine samples. Statistical and bioinformatics methods are used to identify significantly different metabolites that can discriminate CHD cases from healthy controls. Hierarchical cluster analysis (HCA) is performed to identify metabolites clusters contributing to phenotype separation and spearman correlation analysis is applied to identify potential biomarkers’ correlations related to abnormal functions. Pathway analysis and association analysis of potential biomarkers and gut flora are then applied. Finally, potential biomarkers associated gut flora species are discovered. Metagenomics technology is applied to further validate the potential metabolites originated from the fecal metagenomics data of CHD patients and healthy subjects.

The detailed workflow for metabolomics and metagenomics study was illustrated in Fig. 1. A total of 1347 peaks (93.67% in original total peaks) and 2858 peaks (96.68% in original total peaks) were obtained in plasma and urine samples respectively after quality control. The stability and reproducibility of current data was evaluated by the QC samples measured during the whole experimental period. Principle component analysis (PCA) scores plot representation of QC samples for plasma and urine samples were shown in Supplementary Fig. S1a and Fig. S1b respectively. No drift in the metabolites profiles obtained in positive ion modes, were observed demonstrating good stability and reproducibility in our current metabolomics data set.

### Metabolic findings in Plasma Samples

**Figure 2:**
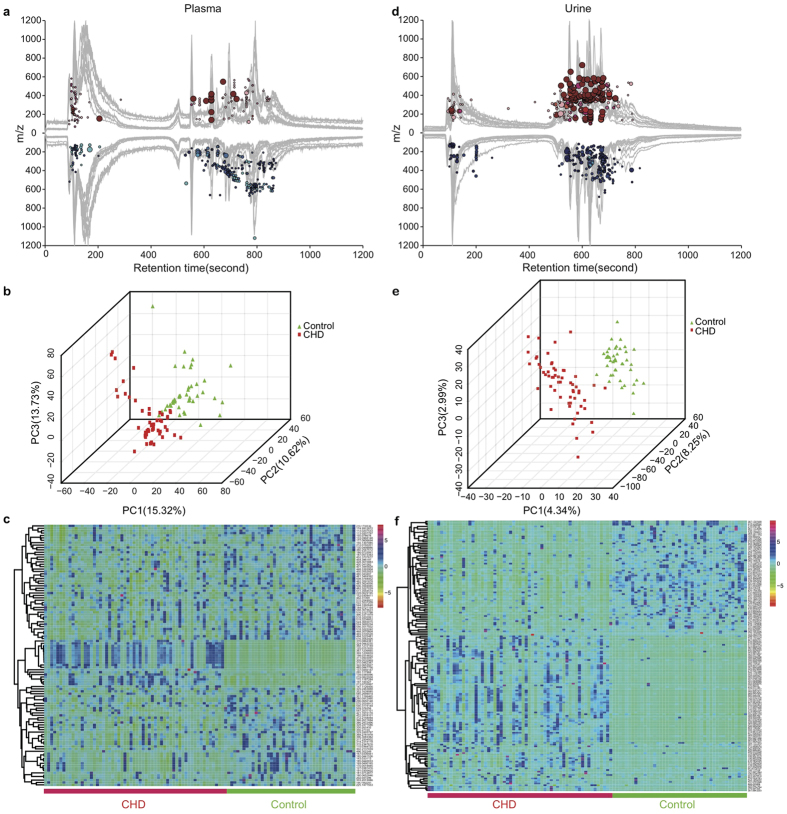
Potential biomarkers discovery in plasma and urine metabolomics. (**a**) Cloud plot of plasma metabolites profiles demonstrated significant metabolic changes had happened in CHD patients’ plasma. Red and blue circles indicated metabolites with increased (*fold change* > 1.2, 196 metabolites) and decreased intensity (*fold change* < 0.8, 319 metabolites) in CHD patients’ plasma samples compared with healthy controls. The darkness of color is correlated with adjusted *p.value* (named as *q.value*)*: c*olor from pink to dark red or cyan to dark blue indicated smaller adjusted *p.value*. The area of circle is correlated with magnitude of intensity change: In the red part, the bigger the circle was, the more enriched metabolites were in CHD patients’ plasma samples compared with healthy controls’ plasma samples. While in the blue part, the bigger the circle was, the more enriched metabolites were in healthy controls’. (**b**) Three-dimensional PLS-DA scores plot of plasma samples. It depicted obvious difference between CHD patients’ plasma samples and healthy controls’ plasma samples with *PC1(15.32%), PC2(10.62%), PC3(13.73%)*. (**c**) Heat map showed the distribution of 109 metabolites that were significantly different between CHD patients’ plasma samples and healthy controls’ plasma samples. The CHD patients’ and healthy control group’s plasma samples were labeled with red and green ribbons and texts respectively. The mass data (m/z) which could be annotated with database such as HMDB, KEGG were listed. (**d**) Cloud plot of urine metabolites profiles also demonstrated significant metabolic changes happened in CHD patients’ urine. (**e**) Three-dimensional PLS-DA scores plot of urine samples with *PC1(4.34%), PC2(8.25%), PC3(2.99%)*. (**f**) Heat map analysis of 160 significantly different metabolites in the urine samples of CHD group and healthy control group.

For plasma samples, cloud plot analysis of the total 1347 peaks (Fig. 2a) showed that the intensity of 196 peaks (14.55%) were increased in CHD patients’ plasma samples (*fold change* > 1.2) while the intensity of 319 peaks (23.68%) were decreased in CHD patients’ (*fold change* < 0.8). Both PCA scores plot (Supplementary Fig. S2a) and three-dimensional partial least squares – discriminant analysis (PLS-DA)^11^ scores plot (Fig. 2b) of these plasma samples showed that there were significant differences between 59 CHD patient samples and 43 healthy control samples. CHD patients’ plasma samples were apart from healthy control’s samples with *PC1, PC2, PC3 as 15.32%, 10.62%, 13.73%* respectively. The permutation multivariate analysis of variance (PERMANOVA) (Supplementary Fig. S2b) was implemented to test the relation of individual’s phenotypes with their metabolite characteristics, and we found CHD status had significant impacts on the metabolic profiling (*p.value* < *0.001*, 1000 permutations) in positive ion mode.

S-plot analysis was used for selection of potentially interesting metabolites biomarkers^12^. Using the criteria that variable importance in the projection (*VIP*) was larger than 1, 230 variables were selected (Supplementary Fig. S2c) in S-plot. On the condition that *adjusted p.value* < 0.05, *fold change* > 1.2 or < 0.8, 414 variables were retained in Volcano-plot (Supplementary Fig. S2d). Combing these two results, 202 shared peaks were obtained (Supplementary Fig. S2e). And a total of 109 significant peaks from these 202 shared peaks could be annotated by aligning the exactly significant peaks’ molecular mass data (m/z) with online database: HMDB and KEGG.

The intensities of 109 annotated metabolites (20 increased and 89 decreased in CHD patients) were included in Supplementary Data S1. The heatmap exhibited the different distribution patterns of metabolites between CHD group and control group (Fig. 2c).To further identify potential metabolites from 109 m/z, both HMDB and HMDB SERUM databases were searched using accurate mass and mass spectrometric fragmentation patterns^13^. We found 18 matched metabolites from the above database, including 13 Lysophosphatidylcholine (LPCs), 2 glycerophosphocholines, L-Arginine, N-Acetyl-D-glucosamine 6-phosphate (GlcNAc-6-P) and paraxanthine (as listed in Table 1). The intensity of 13 LPCs and 2 glycerophosphocholines were lower in CHD patients (as shown in Supplementary Fig. S3a). Besides, the level of L-Arginine and GlcNAc-6-P increased by 2.14 and 8.58 folds in CHD patients. In addition, the level of paraxanthine was significantly decreased in CHD patients.

To evaluate the interaction among these 18 metabolites, spearman correlation analysis was performed. Several metabolites pairs showed relatively strong positive correlations: 1-Oleoylglycerophosphocholine vs LysoPC(20:4(5Z,8Z,11Z,14Z)) (*rho* = *0.929, q.value* = *0*), 1-Palmitoylglyceropho-sphocholine vs LysoPC(18:3(9Z,12Z,15Z)) (*rho* = *0.874, q.value* = *0*), and 1-Oleoylglycerophosphocholine vs 1-Palmitoylglycerophosphocholine (*rho* = *0.748, q.value* = *0*) as shown in Supplementary Fig. S3(b–e), and Supplementary Data S2.

To investigate latent relationships of those 109 significantly changed metabolites, spearman correlation analysis was also performed. As illustrated in Supplementary Fig. S3f, significantly changed plasma metabolites with smaller *adjusted p.value* either in CHD enriched metabolites or in control enriched metabolites had a relatively stronger correlation. Similar to correlation analysis of 13 LPCs and 2 glycerophosphocholine metabolites, analysis among those 18 identified metabolites showed that LysoPC(18:0) had strong positive correlations with the following metabolites: LysoPC(18:0) vs LysoPC(P-16:0) (*rho* = *0.861, q.value* = *0*), LysoPC(20:3(5Z,8Z,11Z)) (*rho* = *0.831, q.value* = *0*), LysoPC(0:0/18:0) (*rho* = *0.802, q.value* = *0)*. LysoPC(16:1(9Z)) had strong positive correlations with LysoPC(14:0) (*rho* = *0.854, q.value* = *0*) and LysoPC(18:0) (*rho* = *0.815, q.value* = *0*). On the other hand, L-Arginine negatively correlated with 1-Palmitoylglycerophosphocholine (*rho* = *−0.558, q.value* = *1.07E-08*).

### Metabolic findings in Urine Samples

In the urine cloud plot (Fig. 2d), there were 870 peaks (30.44%) with increased intensity in CHD patients (*fold change* > *1.2*) while the level of 557 peaks (19.49%) were decreased (*fold change* < *0.8*). PCA and PLS-DA models were used and the analysis results were shown in PCA scores plot (Supplementary Fig. S4a) and three-dimensional PLS-DA scores plot (*PC1(4.34%), PC2(8.25%), PC3(2.99%)*) (Fig. 2e). These results indicated urine metabolic profiles in the CHD patients were significantly different from those in healthy subjects. PERMANOVA analysis (Supplementary Fig. S4b) demonstrated CHD had a significant impact on metabolic profile. Furthermore, S-plot analysis (Supplementary Fig. S4c) and Volcano-plot analysis (Supplementary Fig. S4d) were applied for potential biomarkers discovery. Using these criteria (*VIP* > 1, *adjusted p.value* produced by Mann−Whitney−Wilcoxon test after FDR correction < 0.05, *fold change* > 1.2 or <0.8), 391 peaks were found to be significantly changed in CHD group by intersection of 559 peaks and 558 peaks in S-plot and Volcano-plot, respectively, as is shown in Veen plot (Supplementary Fig. S4e).

The 391 peaks were aligned and annotated using the HMDB and KEGG database. Among the 160 annotated metabolites, the intensities of 96 metabolites were increased while the intensities of the other 64 metabolites were decreased in CHD patients (the intensity data is provided in Supplementary Data S3). These 160 metabolites were used to perform phenotype analysis for the 102 samples. As shown in the heat map (Fig. 2f), the CHD patients’ metabolism was obviously different from healthy controls. By comparing MS/MS spectra and retention time with commercially available reference standards, 4 metabolites were verified and the results were listed in Table 2. The level of GlcNAc-6-P and mannitol were increased with fold change of 165.99 and 8.45 in CHD patients respectively. Meanwhile, the level of creatine and phytosphingosine were decreased with fold changes of 0.41 and 0.39 respectively.

To evaluate correlation among 160 annotated urine metabolites, spearman correlation analysis was performed. The results were shown in Supplementary Fig. S4f. Urine metabolites which were significantly changed (with smaller *adjusted p.value)* had relatively stronger correlations compared with plasma significant metabolites. In addition, among those 4 validated metabolites, mannitol showed a relatively high positive correlation with GlcNAc-6-P (*rho* = *0.775, q.value* = *9.40E-21*).

### Correlations between plasma and urine significant metabolites

**Figure 3:**
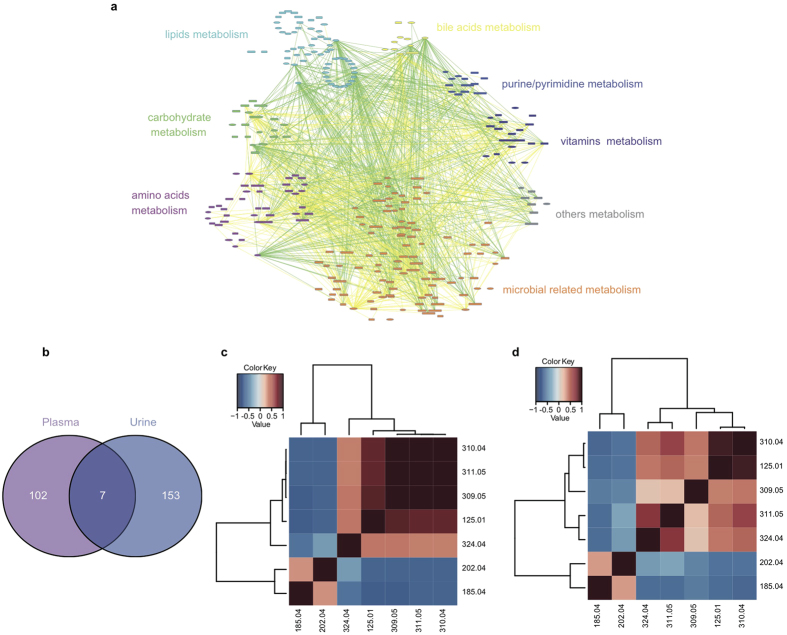
Correlation analysis of all significant metabolites or seven common metabolites in plasma and urine. (**a**) Correlation profile of 109 plasma significant metabolites and 160 urine significant metabolites among CHD samples and control subjects were performed by spearman correlation analysis with Cytoscape software. All these annotated metabolites were distributed by their engaged pathways and metabolisms: lipids metabolism showed significantly negatively correlations with microbial related metabolism. Ellipses were plasma metabolites, round rectangles were urine metabolites. Yellow lines : *0.9* > *rho ≥ 0.5*, Green lines : *rho* ≤ *−0.5*. (**b**) Veen diagram of all significant differential metabolites in plasma and urine showed there are 7 common significantly changed metabolites. (**c**) Spearman correlation analysis of 7 metabolites in plasma. (**d**) Spearman correlation analysis of 7 metabolites in urine.

To illustrate the potential physiological function and build biologic networks of differentially expressed metabolites in plasma and urine^14^, Cytoscape software (3.0.2) was applied to profile the correlations among these significantly changed plasma and urine metabolites. As seen in Fig. 3a, these 109 annotated significantly changed plasma metabolites and 160 annotated significantly changed urine metabolites were involved in different pathways and can be divided into 8 categories: carbohydrate metabolism, lipids metabolism, amino acids metabolism, bile acids metabolism, purine/pyrimidine metabolism, vitamins metabolism, microbial related metabolism and others. Lipids metabolism showed significantly negatively correlations with microbial related metabolism while other 6 metabolism categories were in strong positive correlation with microbial related metabolism, which indicated that microbial may play an important role in the metabolism in CHD.

Seven significantly differential expressed metabolites (Supplementary Table S2), including GlcNAc-6-P, were found both in plasma and urine on the condition that retention time error was less than 1 min and m/z error was less than 0.01 Dalton with MS/MS comparison. A Veen diagram exhibiting the common metabolites among plasma and urine significantly changed metabolites is provided in Fig. 3b. Two metabolites (m/z: 185.04, 202.04) were decreased in CHD patients while other five metabolites (m/z: 125.01, 309.05, 310.04, 311.05, 324.04) were increased in CHD patients.

To evaluate the correlation among 7 common metabolites, spearman correlation analysis was implemented using the criteria that the coefficient was larger than 0.90 (Supplementary Data S4). First, correlation among plasma metabolites was shown in Fig. 3c. m/z 311.05 showed strong correlation with m/z 309.05 (*rho* = *0.929, q.value* = *9.31E-45*), m/z 310.04 (*rho* = *0.911, q.value* = *5.70E-40*), m/z 324.04 (*rho* = *0.900, q.value* = *1.53E-37*); m/z 309.05 also strongly correlated with m/z 310.04 (*rho* = *0.929, q.value* = *9.31E-45*), as shown in Fig. 3c. Second, correlations among urine metabolites were depicted in Fig. 3d, GlcNAc-6-P (m/z 324.04) was strongly correlated with m/z 310.04 (*rho* = *0.933, q.value* = *1.26E-45*), m/z 311.05 (*rho* = *0.910, q.value* = *1.17E-39*), m/z 125.01 (*rho* = *0.903, q.value* = *3.43E-38*), while m/z 125.01 also showed strong correlation with urine metabolite (m/z 310.04 (*rho* = *0.918, q.value* = *1.28E-41*). In addition, correlation of these metabolites in plasma and urine was also evaluated. The results showed that plasma metabolite have strong positive correlations with the same metabolites in urine (Supplementary Table S3). Among them, validated GlcNAc-6-P (324.04) showed very strong positive correlation with itself (*rho* = *0.747, q.value* = *5.60E-19*).

### Clinical relevance of plasma and urine potential metabolites

#### Receiver operating characteristic analysis

To evaluate the potential of the identified metabolites(18 plasma and 4 urine ones) as biomarkers, receiver operating characteristic analysis (ROC) was applied to 176 additional plasma samples (98 controls vs78 CHD patients) and 395 additional urine samples (173 controls vs 222 CHD patients).

**Figure 4:**
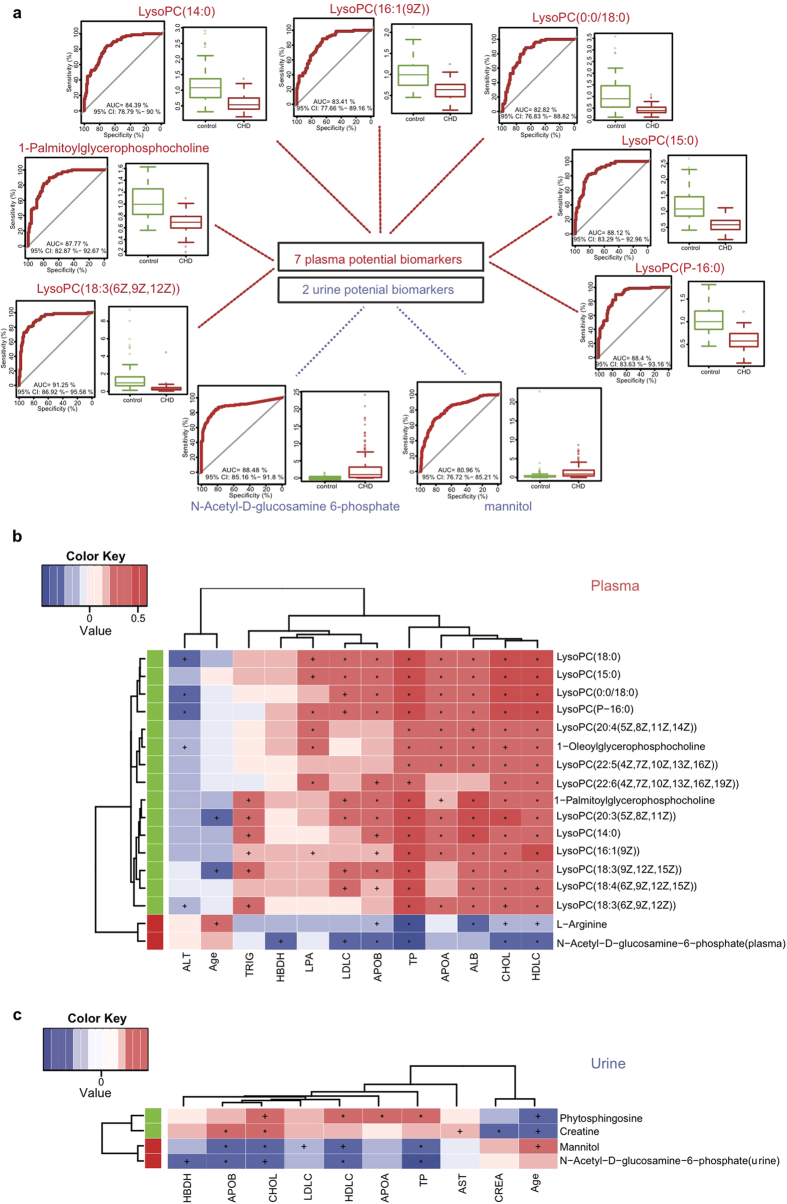
Receiver operating characteristic (ROC) analysis of potential biomarkers and numeric correlation between clinical phenotype and identified significant metabolites. (**a**) ROC analysis and boxplots of 7 identified plasma potential biomarkers and 2 identified urine potential biomarkers with *AUC* larger than 0.80 in validation datasets. (**b**) Spearman correlation analysis was performed between 18 plasma identified potential biomarkers and clinical indicators. Red, positive correlation; blue, negative correlation. + , adjusted *p.value* < 0.05; *, adjusted *p.value* < 0.01. Red panel indicated increased metabolites in CHD patients while green panel suggested decreased metabolites in CHD patients. Paraxanthine did not show significant correlations with any of the 15 numerical phenotypes (adjusted *p.value* > 0.05, Spearman’s), creatine kinase MB (CKMB), aspartate transaminase (AST) and creatinine (CREA) did not show significant correlations with any of 18 plasma identified potential biomarkers, both of which were not shown. albumin (ALB), alanine aminotransferase (ALT), total protein (TP), hydroxybutyrate dehydrogenase (HBDH), triglyceride (TRIG), low-density lipoprotein (LDLC), cholesterol (CHOL), high-density lipoprotein (HDLC), apolipoprotein (**b**) (APOB), apolipoprotein (**a**) (APOA), lipoprotein (**a**) (LPA). (**c**) Spearman correlation analysis was performed between 4 urine identified potential biomarkers and clinical indicators. CKMB, ALB, ALT, TRIG and LPA did not show significant correlations with any of 4 urine identified potential biomarkers were not shown.

In plasma validation datasets, 6 LPCs and 1 glycerophosphocholine metabolites showed area under curve (*AUC*) larger than 0.80 and were significantly different in CHD patients (Table 3). As shown in Fig. 4a, The levels of LysoPC(18:3(6Z,9Z,12Z)), LysoPC(P-16:0), LysoPC(15:0), 1-Palmitoylglycerophosphocholine, LysoPC(14:0), LysoPC(16:1(9Z)), LysoPC(0:0/18:0) were decreased in CHD patients with *fold change* at 0.26, 0.58, 0.51, 0.65, 0.49, 0.62, 0.42 respectively and *AUC* of 0.91, 0.88, 0.88, 0.88, 0.84, 0.83, 0.83 respectively. On the other hand, other 9 plasma potential biomarkers exhibited the same enrichment direction except that LysoPC(20:3(5Z,8Z,11Z)) became normal and GlcNAc-6-P even became undetected (data shown in Table 3, the training datasets ROC shown in Supplementary Fig. S5 and the validation datasets ROC shown in Supplementary Fig. S6a–k). These results suggested that LPCs could become biomarkers and targets for CHD diagnosis and therapies in the future.

In urine validation datasets, GlcNAc-6-P and mannitol exhibited *AUC* of 0.88, 0.81 and *fold change* at 36.91 and 2.62 respectively (as shown in Fig. 4a and Table 4). However, creatine and phytosphingosine did not show good diagnostic ability in both training and validation datasets (Supplementary Fig. S6l, m). The ROC of training datasets was shown in Supplementary Fig. S7.

Among these 7 choline metabolites and 2 urine metabolites with *AUC* larger than 0.80, GlcNAc-6-P appeared the most discriminative biomarker which showed relatively good diagnostic ability with false negative (*FN*) of 0.051, 0.153 and false positive (*FP*) of 0.047, 0.208 in the training datasets and validation datasets respectively. LysoPC(18:3(6Z,9Z,12Z)), LysoPC(P-16:0), LysoPC(15:0), 1-Palmitoylglycerophosphocholine, LysoPC(14:0), LysoPC(16:1(9Z)), LysoPC(0:0/18:0) and mannitol exhibited diagnostic ability with *FN* of 0.271, 0.169, 0.136, 0.068, 0.119, 0.119, 0.085, 0.153 and *FP* of 0.233, 0.163, 0.256, 0.233, 0.209, 0.140, 0.279, 0.093 in the training datasets; Meanwhile, in the validation datasets, their *FN* were 0.013, 0, 0, 0.013, 0.013, 0.013, 0, 0.135 and *FP* were 0.582, 0.755, 0.673, 0.694, 0.612, 0.684, 0.714, 0.416 respectively.

#### Association of potential metabolic biomarkers with clinical phenotypes

To access the effects of patients’ covariates (such as age and clinical biochemical factors) on metabolic profiles, PERMANOVA analysis was performed. Albumin (ALB, permuted *p.value* = *8.40E-03*), alanine aminotransferase (ALT, permuted *p.value* = *0.02*), total protein (TP, permuted *p.value* = *1.00E-04*), low-density lipoprotein (LDLC, permuted *p.value* = *0.01*), cholesterol (CHOL, permuted *p.value* = *1.00E-04*), high-density lipoprotein (HDLC, permuted *p.value* = *1.00E-04*), apolipoprotein b (APOB, permuted *p.value* = *5.00E-04*) and apolipoprotein a (APOA, permuted *p.value* = *8.10E-03*) were found to be significantly different in CHD patients (Supplementary Table S1). Together with the results from clinical phenotypes student t-test, PERMANOVA analysis again proved that these clinical phenotypes showed significant effects on the plasma and urine metabolic profile in CHD patients differing from healthy controls.

Besides, spearman correlation analysis was performed among 18 potential plasma biomarkers (Fig. 4b) and 4 potential urine biomarkers (Fig. 4c) with individual phenotypes. CHOL, HDLC and TP showed significantly positive correlation with plasma LPCs (Supplementary Table S4).

LysoPC (18:0) was correlated with CHOL (*rho* = *0.518, q.value* = *7.89E-07*), HDLC (*rho* = *0.548, q.value* = *1.29E-07*) and TP (*rho* = *0.573, q.value* = *5.16E-08*). LysoPC(P-16:0) was positively correlated with HDLC (*rho* = *0.561, q.value* = 7.39E-08). This result showed that LPCs metabolism is significantly abnormal in CHD patients, and thus we speculated that it could be beneficial to reduce CHD occurrence by properly increasing intake of these extra LPCs which were significantly decreased in CHD patients. Meanwhile, the two potential urine biomarkers, GlcNAc-6-P and mannitol, exhibited strong negative correlations with CHOL, HDLC, TP and APOB (*q.value* < 0.01).These results confirmed GlcNAc-6-P worked as a negative effector and may influence the normal metabolic processes in our body, and could be used as a good biomarker for CHD. The level of GlcNAc-6-P level in urine should be monitored closely for tracking CHD status.

### Gut flora associated potential metabolite biomarkers

**Figure 5:**
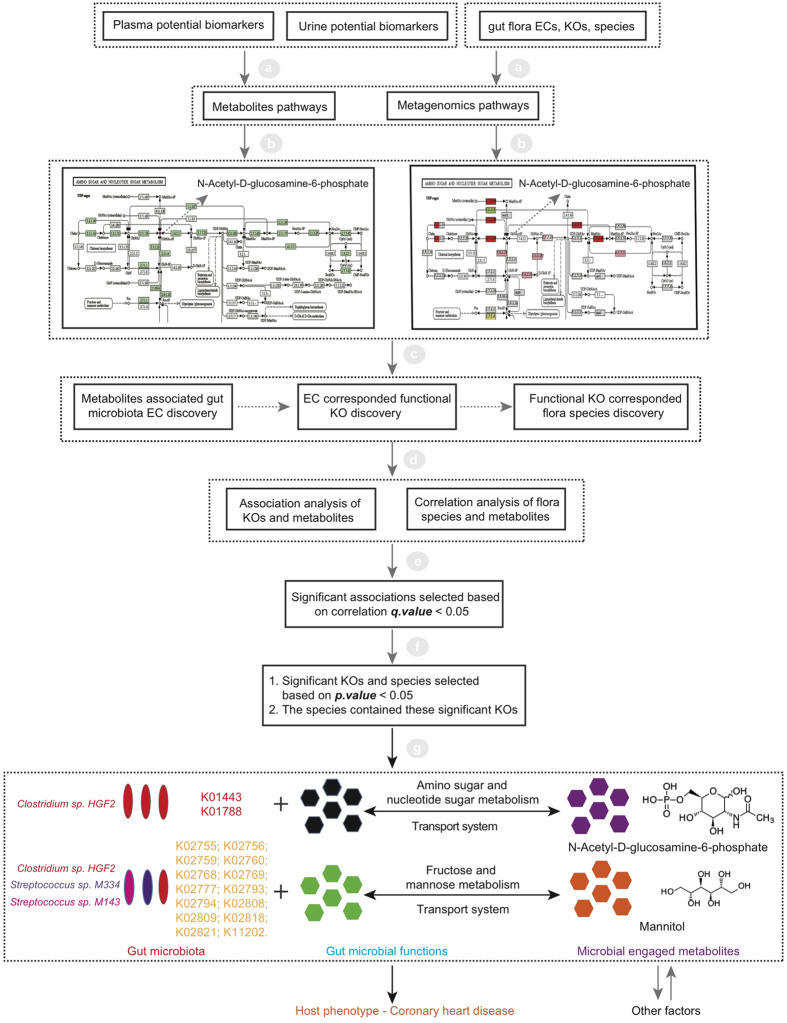
A workflow for the discovery of interactions between metabolites and gut microbiota. Pathways analysis and association analysis among plasma, urine potential biomarkers and gut microbiota were implemented in the workflow. First, plasma and urine potential biomarkers could be obtained in the previous metabolomics studies, the information of gut flora ECs, KOs and species could be attained in the metagenomics study. They could be applied for the metabolic and metagenomics pathways constructions. Second, we could find the metabolites corresponded ECs by analysing the metabolic and metagenomics pathways and get the corresponded KOs by tracing the ECs data, further we could obtain the corresponded species by tracing the KOs data. Third, association analysis would be performed between KOs and metabolites, species and metabolites. Significant correlations would be obtained on the condition of correlation *q.valu*e < 0.05. Lastly, in these significant correlations, we further strictly screened these correlations on the conditions that the correlated KOs and species should be significant in the metagenomics data (*p.value* < 0.05), and the correlated species should contain these significantly correlated KOs. By integrating these metabolomics and metagenomics data, *Clostridium sp. HGF2* was found to significantly correlate with GlcNAc-6-P. *Clostridium sp. HGF2*, *Streptococcus sp. M143*, *Streptococcus sp. M334* were found to significantly associate with mannitol.

Human body is a complex biosystem with numerous co-existing microbial species. Previous study suggests that around 30% of metabolites detected in human body originate from microbiota^15^. In the amino sugar and nucleotide sugar metabolism pathway, it shows that GlcNAc-6-P could be produced by human body enzymes and gut bacterial enzymes. The facts that no related *homo sapiens* enzymes are found in the fructose and mannose metabolism so far indicate mannitol might belong to microbial metabolites family, and current reports suggest it could be produced by several microorganisms such as *lactic acid bacteria*^16^ and *pseudomonas putida*^17^. Pathway analysis for plasma and urine metabolites indicates that some potential biomarkers like GlcNAc-6-P and mannitol might be of microbial origin. To discover gut flora species significantly associated with the identified potential biomarkers, integrated analysis of metabolomics and metagenomics were performed for the patient and control groups, as shown in Fig. 5, and the annotated 512 mOTU species profile was provided in the Data S5 and the analysis results of the differences among these 512 mOTU species in 102 samples were included in the Data S6.

Analysis of metabolites pathway suggested that the following 4 metabolic pathways were significantly changed in CHD patients compared with healthy controls: amino sugar and nucleotide sugar metabolism, arginine and proline metabolism, glycerophospholipid metabolism, fructose and mannose metabolism. Integration of metabolic and metagenomic pathways showed that gut-related microbial metabolites such as GlcNAc-6-P, mannitol, creatine, and LPCs, were involved in CHD pathways.

The corresponding gut microbial EC affecting the productions and functions of those significantly changed potential biomarkers were analyzed. The significantly changed ECs associated with GlcNAc-6-P were EC(2.7.1.69), EC(2.7.1.59), EC(3.2.1.14), EC(5.1.3.9), EC(2.7.1.60), EC(3.5.1.25), EC(5.4.2.10), EC(2.3.1.157), EC(2.7.7.23) and EC(4.1.3.3). Meanwhile, EC(3.5.2.10) was associated with creatine ; EC(1.1.1.14), EC(2.7.1.69) and EC(3.2.1.80) was associated with mannitol; EC(3.5.3.6), EC(2.1.3.3), and EC(2.1.3.9) was associated with arginine; EC(3.1.1.5), EC(3.1.1.32) was correlated with LPCs.

These significantly changed ECs were then annotated with 65 KOs. And spearman correlation analysis was applied to these 65 KOs and the total 22 identified potential plasma and urine metabolites biomarkers (18 plasma ones and 4 urine ones listed in Tables 1 and 2). The results showed that 16 CHD enriched KOs were significantly correlated with GlcNAc-6-P (both in plasma and urine) and mannitol (in urine) (Supplementary Table S5).

To further identify the microbial species correlated with the 22 significantly changed metabolites, spearman correlation analysis of those 22 biomarkers with 512 annotated species was also implemented (Table 5). One gut flora species – *Clostridium sp. HGF2* (*p.value* = 9.86E-05, *q.value* = 8.65E-03), was found to positively significantly correlate with GlcNAc-6-P in amino sugar and nucleotide sugar metabolism and transport system pathways. Meanwhile, 3 gut flora species - *Streptococcus sp. M334* (*p.value* = 3.13E-02, *q.value* = 2.39E-01), *Streptococcus sp. M143* (*p.value* = 3.49E-02, *q.value* = 2.40E-01), *Clostridium sp. HGF2* (*p.value* = 9.86E-05, *q.value* = 8.65E-03) were positively correlated with mannitol in fructose and mannose metabolism and transport system pathways. These three gut flora species were CHD enriched gut microbiota. Interestingly, *Clostridium sp. HGF2* positively associated with both GlcNAc-6-P and mannitol.

In summary, by combining the association results of KOs and flora species with those 22 identified potential biomarkers, we found that *Streptococcus sp. M334* and *M143*, *Clostridium sp. HGF2* and their associated metabolites GlcNAc-6-P and mannitol were involved in the development of coronary heart disease. This study provides the first direct evidence that microbial metabolites are involved in the CHD disease. Besides, the corresponding flora species regulating the microbial metabolites in plasma and urine of CHD patients are identified.

## Discussion

In this study, MS-based metabolomics approach was applied to study the metabolic phenotype variations between CHD patients (n = 53) and healthy controls (n = 49) with complementary metagenomics technology for bacterial metabolites associated intestinal flora discovery. Among these 59 CHD patients, 32 patients had undergone Percutaneous Coronary Intervention (PCI) before but no difference had been observed between these 32 postoperative patients group and those 27 patients group with no surgery (as shown in PCA scores plots in Fig. S8), suggest the PCI did not influence the whole metabolic pattern in patients. However, some conditions were not taken into considerations in our study, such as age, gender, BMI, pre-clinical treatments (medications for hypertension, nonsteroidal anti-inflammatory drug (NSAID) use, prescribed lipid-lowering drugs etc), cardiovascular disease history (heart disease or stroke), physical activity, special diet, dietary supplement use, total energy intake per day (kcal/day), cigarette smoking, sleeping, education, overweight, obesity, family genetics.

In plasma, 18 significantly changed metabolites (13 LPCs, 2 glycerophosphocholines, L-Arginine, GlcNAc-6-P and paraxanthine) were identified as potential biomarkers. LPCs are the major components of ox-LDL which show dual functions in the cardiovascular disease. It could accelerate the formation and development of atherosclerosis by promoting cell proliferation, enhancing lymphocyte adhesion, differentiation and activation^18^,^19^,^20^. GlcNAc-6-P is an endogenous metabolite which could be synthesized and metabolized by amino sugar and nucleotide sugar metabolism^21^. GlcNAc-6-P could be converted into glucosamine-6-P by N-acetylglucosamine-6-phosphate deacetylase (NagA) enzyme which plays a central role in cell wall synthesis and glycolysis, and its intermediate metabolite N-acetylglucosamine-6-P (GlcN-6P) would be metabolized to fructose-6-P (Fru-6P) by glucosamine-6-phosphate deaminase for futher glycolysis or gluconeogenesis. Paraxanthine is a preferential metabolite of caffeine in caffeine metabolism in animals, a psychoactive central nervous system (CNS) stimulant and competitive nonselective phosphodiesterase inhibitor^22^. In urine, 4 significantly changed metabolites (GlcNAc-6-P, mannitol, creatine, phytosphingosine) were identified as potential biomarkers in CHD patients. Changed mannitol in human body could induce water and electrolyte disorders in CHD patients. High level of mannitol in the human body could rapidly increase blood volume, cause diluted hyponatremia or accidentally hyperkalemia and even lead to heart failure. Creatine was also found to be decreased in CHD patient’s urine sample, a nitrogenous organic acid naturally produced by the human body from amino acids. In biosystem, creatine can elevate creatine phosphate levels and improve maintenance of ATP content during tissue oxygen depletion period, and it also has the capacity to scavenge free radicals and reduce oxidative stress^23^. Phytosphingosine is a phospholipid and a major component of mammalian tissue biological membranes. The synthesis of phytosphingosine can be performed by human body and intestinal microbiota in the sphingosine metabolism. Phytosphingosine could induce caspase-independent apoptosis in human T-cell lymphoma and non-small cell lung cancer cells^24^,^25^. Among these 22 metabolites evaluated by ROC, GlcNAc-6-P appears the most discriminative biomarker which shows relatively good diagnostic ability with *FN* of 0.153 and *FP* of 0.208. Correlation analysis between potential biomarkers and biochemical clinical data suggest plasma LPCs are significantly positive correlated with cholesterol (CHOL), high-density lipoprotein (HDLC), and total protein (TP), while GlcNAc-6-P and L-arginine exhibit negative correlations with CHOL, HDLC, and TP. This suggests the metabolites may potentially influence the normal metabolic pathways in our body.

As estimated, over 30% of metabolites in human body originate from intestinal microbes and may contribute to host diseases^15^. In this study, metabolomics and metagenomics techniques were integrated and evidence that microbial species and their associated metabolites were involved in CHD diseases were uncovered for the first time. Firstly, mannitol was identified as a potential urine biomarker in CHD patients. The fact that no related homo sapiens enzymes are found in the fructose and mannose metabolism so far indicates mannitol should belong to microbial metabolites family. Mannitol was previously reported to be produced by *lactic acid bacteria*^16^ and *pseudomonas putida*^17^. In current study, spearman correlation analysis of, KOs, species and mannitol indicates that three gut flora species, *Clostridium sp. HGF2*, *Streptococcus sp. M334*, and *Streptococcus sp. M143*, play important roles in metabolism of mannitol. This was further validated by mannose-specific IIB component of PTS system (EC:2.7.1.69) was found to be the common enzyme in all three CHD enriched gut microbiota species. Secondly, GlcNAc-6-P, an endogenous and microbial metabolites, was identified in both plasma and urine samples of CHD patients. GlcNAc-6-P participates in sugar metabolism with dual functions in regulating host cardiovascular activity. In our study, the metabolism of GlcNAc-6-P was found to be significantly correlated with *Clostridium sp. HGF2* by NagA (EC:3.5.1.25) and N-acylglucosamine-6-phosphate 2-epimerase (EC:5.1.3.9). The discovery of these two microbial metabolites (Mannitol and GlcNAc-6-P) and their correlated microbiota in CHD patients has two important implications. First, it confirmed that microbial metabolites can be used as potential biomarkers for CHD diagnosis along with other traditional metabolites. For instance, GlcNAc-6-P in urine exhibited relatively strong CHD diagnostic ability with *AUC* of 0.88 and showed *FN* of 0.153 and *FP* of 0.208 in the ROC analysis of validation datasets. Second, microbial metabolites reflect the abnormalities of the host intestine microbiota, so new strategy for CHD treatments can be developed by adjusting patients’ gut intestine ecosystem. In the future, microbial species and their associated metabolites could be used as new indexes and targets for diagnosis and treatment of CHD.

In summary, this work had demonstrated significantly altered metabolisms and metabolites, especially gut microbiota related metabolites and metabolites significantly positively associated gut flora species, in the CHD patients compared with healthy controls through MS-based metabolomics and metagenomics technology, providing targets for CHD dynamic detection and monitoring. The findings in current study could be further validated and investigated in several ways. First, a larger number of plasma, urine and fecal samples are needed for population-based validation; Second, semi-quantitative method was used for LC-MS data collection analysis in current paper. For future clinical applications, absolute quantitative analysis is recommended for stable and reliable biomarkers detection and monitoring. Lastly, two metabolites GlcNAc-6-P and mannitol were found to be significantly positively associated with gut microbiota such as *Clostridium sp. HGF2*, *Streptococcus sp. M334* and *Streptococcus sp. M143*. The underlying mechanisms regulating correlation between gut microbiota and metabolites like GlcNAc-6-P and mannitol in CHD incidents could be further investigated.

## Materials and Methods

### Clinical samples

All patients with CHD diagnosed by coronary angiography techniques were recruited from the Guangdong General Hospital. All control people enrolled in our study were free of clinically evident coronary artery disease (CAD) at medical examination during the same period.

Paired plasma, urine and fecal samples of CHD patients (n = 59) and healthy controls (n = 43) were obtained from the Guangdong General Hospital on the same day. Coronary angiography techniques were performed to diagnose CHD patients recruited in this study. The healthy controls had underwent physical examination in the same hospital. Patients and controls did not receive probiotics or antibiotics within one month before sample collection. Among these 59 CHD patients, 32 patients had undergone Percutaneous Coronary Intervention (PCI) before. The participants’ clinical information was provided in Supplementary Table S1. Besides, 176 additional plasma samples (98 controls vs 78 CHD patients) and 395 additional urine samples (173 controls vs 222 CHD patients) were included for potential biomarkers diagnostic capability analysis, while another 314 fecal samples (155 controls vs 159 CHD patients) were included for gene catalogue construction.

The details of samples collections, samples preparations for HPLC-MS experiments, infrastructure parameters of HPLC-MS experiments, DNA extraction from fecal samples, DNA library construction and metagenomics sequencing of fecal samples, and experiments related materials could be found as Supplementary Materials and Methods. All these protocols were reviewed and approved by the Institutional Review Board of BGI-Shenzhen. Before collecting samples, patients were informed and written consent were obtained from them. Plasma, urine and fecal sampling and studies were carried out according to the approved protocols and guidelines.

### HPLC-MS data analysis

The acquired MS data pretreatments including peak picking, peak grouping, retention time correction, second peak grouping, and annotation of isotopes and adducts was performed using the same method as our previously published work^26^. LC−MS raw data files were converted into *mzXML* format and then processed by the XCMS and CAMERA toolbox implemented with the R software (*v3.1.1*). Each ion was identified by combining retention time (RT) and m/z data. Intensities of each peaks were recorded and a three dimensional matrix containing arbitrarily assigned peak indices (retention time-m/z pairs), sample names (*observations*) and ion intensity information (*variables*) was generated.

The obtained matrix was further reduced by removing peaks with more than 80% missing values (*ion intensity* = *0*) and those with isotope ions from each groups in order to obtain consistent results. As a quality assurance strategy in metabolic profiling, all retained peaks were normalized to the QC sample using Robust Loess Signal Correction (R-LSC) based on the periodic analysis of a standard biological quality control sample (QC sample) together with the real plasma and urine samples to ensure that the data are of high quality within an analytical run^27^. The relative standard deviation (*RSD*) values of metabolites in the QC samples was set at a threshold of 30% which was accepted as a standard in the assessment of repeatability in metabolomics data sets.

The nonparametric univariate method (Mann−Whitney−Wilcoxon test) was performed to measure and discover the significantly changed metabolites among the CHD patients and control subjects and then corrected by false discovery rate (FDR) to ensure that metabolite peaks were reproducibly detected. And multivariate statistical analysis (PCA, PLS-DA) were performed to discriminate CHD samples from control subjects. A number of metabolites responsible for the difference in the metabolic profile scan of CHD patients and control subjects can be obtained on the basis of variable importance in the projection (*VIP*) threshold of 1 from the 10-fold cross-validated PLS-DA model. The PLS-DA model was validated with permutation multivariate analysis of variance (PERMANOVA), a permutation-based version of the multivariate analysis of variance, which was performed in R using the “vegan” package to test the statistical significant differences between metabolic profiles and individuals’ phenotypes^28^. Three dimensional PLS-DA analysis was also implemented to show the difference between CHD samples and control subjects. By combining the univariate and multivariate statistical analysis, significantly changed metabolites distinguishing CHD patients from control subjects were acquired on the condition of *p.value* < 0.05, *q.value* < 0.05, *fold change* < 0.8 or > 1.2, *VIP* > 1. Phenotype analysis was performed to cluster those significantly distributed metabolites and heatmap was used to depict the relatively disturbed and unbalanced metabolism state among CHD samples and control subjects. Spearman correlation analysis was implemented among those significantly changed plasma metabolites, urine metabolites and clinical data of CHD patients and control subjects and correlations of metabolites was profiled with Cytoscape software 3.0.2. In addition, receiver operating characteristic (ROC) analysis was used to evaluate diagnostic capability of identified potential biomarkers with the online tool - ROCCET (http://www.roccet.ca)^29^.

### Metabolites annotations and identifications

The online HMDB database (http://www.hmdb.ca)^30^,^31^,^32^ and KEGG database (www.genome.jp/kegg/)^33^,^34^ were used to annotate the metabolites by matching the exact molecular mass data (m/z) of samples with those from database. If a mass difference between observed and the database value was less than 10 ppm, the metabolite would be annotated and the molecular formula of metabolites would further be identified and validated by the isotopic distribution measurements. Reference standards were purchased and used to validate and confirm those significantly changed metabolites by comparing their MS/MS spectra and retention time.

### Gene catalogue construction

For the sequencing reads of the 314 samples, the employed parameters were the same as previous publication^35^, de novo assembly and gene prediction was performed using SOAPdenovo v1.06^36^ with specific parameter ‘-M 3’ for metagenomics data and GeneMark v2.7^37^ softwares, respectively. All predicted genes were aligned pairwise using BLAT^38^. Redundant genes were removed using BLAT with the cutoff of 90% overlap and 95% identity (no gaps allowed), resulting in a non-redundant gene catalogue comprising of 4,537,046 genes (4.5 M gene catalogue).

### Taxonomic assignment of genes

Taxonomic assignment of the predicted genes was performed using an in-house pipeline which was described in previous publication^35^, with 80% overlap and 65% identity top 10% scores (BLASTN^39^ v2.2.24, -e 0.01 -b 100 -K 1 -F T -m 8). The cutoffs were 65% identity for assignment to phylum, 85% identity to genus, 95% identity to species and ≥ 50% consensus for the taxon under question, if multiple hits remained.

### Functional annotation

Putative amino acid sequences, which translated from our gene catalogue, were aligned against the proteins/domains in KEGG databases (release 59.0) using BLASTP^39^ (e-value ≤ 1e-5). Each protein was assigned to the KEGG orthologue group (KO) by the highest scoring annotated hit(s) containing at least one HSP scoring over 60 bits.

### Species profiles

Total fecal clean reads were aligned to the 79268 sequences of mOTU reference^40^ with default parameters. 512 species level were identified.

## Additional Information

**How to cite this article**: Feng, Q. *et al*. Integrated metabolomics and metagenomics analysis of plasma and urine identified microbial metabolites associated with coronary heart disease. *Sci. Rep*. **6**, 22525; doi: 10.1038/srep22525 (2016).

## Supplementary Material

Supplementary Information

Supplementary Dataset S1

Supplementary Dataset S2

Supplementary Dataset S3

Supplementary Dataset S4

Supplementary Dataset S5

Supplementary Dataset S6

## Figures and Tables

**Table 1:** Potential plasma biomarkers for discriminating CHD patients from control subjects.

m/z	RT(min)*	FC(CHD/control)†	Adjusted p.value‡	VIP§	Adduction	Formula	Metabolites	Pathways
175.11	1.83	2.14	1.36E-11	1.61	H^+^	C6H14N4O2	L-Arginine||	Arginine and proline metabolism
181.07	9.05	0.43	2.39E-02	1.34	H^+^	C7H8N4O2	Paraxanthine||	Caffeine metabolism
496.33	13.19	0.67	2.25E-11	1.36	NAN	C24H51NO7P	1-Palmitoylglycerophosphocholine||	Unknown
522.35	13.33	0.72	1.02E-07	1.15	NAN	C26H53NO7P	1-Oleoylglycerophosphocholine||	Unknown
324.04	9.33	8.58	2.08E-12	2.16	Na^+^	C8H16NO9P	N-Acetyl-D-glucosamine 6-phosphate||,¶	Amino sugar and nucleotide sugar metabolism
468.30	12.69	0.48	1.30E-10	1.8	H^+^	C22H46NO7P	LysoPC(14:0)||	Glycerophospholipid metabolism
480.34	13.48	0.65	4.12E-10	1.4	H^+^	C24H50NO6P	LysoPC(P-16:0)||	Glycerophospholipid metabolism
482.32	12.92	0.57	1.37E-08	1.58	H^+^	C23H48NO7P	LysoPC(15:0)||	Glycerophospholipid metabolism
494.32	12.82	0.54	6.12E-12	1.74	H^+^	C24H48NO7P	LysoPC(16:1(9Z))||	Glycerophospholipid metabolism
516.31	13.21	0.67	5.12E-07	1.19	H^+^	C26H46NO7P	LysoPC(18:4(6Z,9Z,12Z,15Z))||	Glycerophospholipid metabolism
518.32	13.19	0.78	7.15E-11	1.04	H^+^	C26H48NO7P	LysoPC(18:3(9Z,12Z,15Z))||	Glycerophospholipid metabolism
518.32	12.71	0.57	1.75E-06	1.43	H^+^	C26H48NO7P	LysoPC(18:3(6Z,9Z,12Z))||	Glycerophospholipid metabolism
524.36	14.27	0.54	2.19E-08	1.65	H^+^	C26H54NO7P	LysoPC(18:0)||	Glycerophospholipid metabolism
524.36	13.69	0.61	2.25E-11	1.64	H^+^	C26H54NO7P	LysoPC(0:0/18:0)||	Glycerophospholipid metabolism
544.33	13.34	0.76	2.18E-07	1.02	H^+^	C28H50NO7P	LysoPC(20:4(5Z,8Z,11Z,14Z))||	Glycerophospholipid metabolism
546.35	14.22	0.5	1.11E-09	1.81	H^+^	C28H52NO7P	LysoPC(20:3(5Z,8Z,11Z))||	Glycerophospholipid metabolism
570.35	13.05	0.67	8.07E-05	1.14	H^+^	C30H52NO7P	LysoPC(22:5(4Z,7Z,10Z,13Z,16Z))||	Glycerophospholipid metabolism
590.31	12.86	0.6	9.89E-05	1.31	Na^+^	C30H50NO7P	LysoPC(22:6(4Z,7Z,10Z,13Z,16Z,19Z))||	Glycerophospholipid metabolism

^*^Retention time.

^†^Fold change.

^‡^Adjusted *p.value* calculated by the two-tailed Wilcoxon rank-sum tests after false discovery rate correction.

^§^VIP (Variable Importance for Projection), one indicator reflecting the capability of the variables to explain Y.

^||^Metabolites matched with the online database.

^¶^Metabolites which have matched characteristic peaks but mismatched retention time with commercial available reference standards.

**Table 2:** Potential urine biomarkers for discriminating CHD patients from control subjects.

m/z	RT(min)*	FC(CHD/control)†	Adjusted *p.value*‡	VIP§	Adduction	Formula	Metabolites	Pathways
132.07	1.9	0.41	2.36E-02	1.41	H^+^	C4H9N3O2	Creatine#	Arginine and proline metabolism
205.06	1.79	8.45	9.69E-12	2.87	Na^+^	C6H14O6	Mannitol#	Fructose and mannose metabolism
318.29	11.37	0.39	1.65E-05	2.51	H^+^	C18H39NO3	Phytosphingosine#	Sphingolipid metabolism
324.04	10.25	165.99	4.28E-14	2.3	Na^+^	C8H16NO9P	N-Acetyl-D-glucosamine 6-phosphate¶,#	Amino sugar and nucleotide sugar metabolism

^*^Retention time.

^†^Fold change.

^‡^Adjusted p.value calculated by the two-tailed Wilcoxon rank-sum tests after false discovery rate correction.

^§^VIP (Variable Importance for Projection), one indicator reflecting the capability of the variables to explain Y.

^#^Metabolites matched with commercial available reference standards.

^¶^Metabolites which have matched characteristic peaks but mismatched retention time with commercial available reference standards.

**Table 3:** AUC results of plasma training and validation datasets.

Name	Plasma training datasets			Plasma validation datasets		
	FC(CHD/control)‡	*p.value*†	AUC∗	FC(CHD/control)‡	*p.value*†	AUC∗
LysoPC(18:3(6Z,9Z,12Z))	0.57	2.72E-07	0.79	0.26	6.72E-09	0.91
LysoPC(P-16:0)	0.65	2.35E-11	0.88	0.58	2.57E-21	0.88
LysoPC(15:0)	0.57	6.56E-11	0.85	0.51	6.94E-19	0.88
1-Palmitoylglycerophosphocholine	0.67	4.56E-14	0.91	0.65	1.45E-20	0.88
LysoPC(14:0)	0.48	2.86E-11	0.89	0.49	5.94E-15	0.84
LysoPC(16:1(9Z))	0.54	7.78E-14	0.92	0.62	5.57E-16	0.83
LysoPC(0:0/18:0)	0.61	7.98E-15	0.91	0.42	6.05E-13	0.83
LysoPC(18:4(6Z,9Z,12Z,15Z))	0.67	3.81E-08	0.81	0.55	1.65E-12	0.79
LysoPC(22:6(4Z,7Z,10Z,13Z,16Z,19Z))	0.6	1.30E-05	0.74	0.69	7.34E-10	0.76
L-Arginine	2.14	1.52E-11	0.91	1.64	7.63E-10	0.75
LysoPC(22:5(4Z,7Z,10Z,13Z,16Z))	0.67	4.70E-06	0.74	0.73	4.00E-06	0.73
LysoPC(18:3(9Z,12Z,15Z))	0.78	9.90E-14	0.9	0.77	5.42E-06	0.71
LysoPC(18:0)	0.54	2.98E-10	0.84	0.83	1.54E-06	0.7
1-Oleoylglycerophosphocholine	0.72	5.50E-09	0.83	0.88	1.24E-03	0.64
Paraxanthine	0.43	7.33E-03	0.62	0.33	4.41E-04	0.63
LysoPC(20:4(5Z,8Z,11Z,14Z))	0.76	1.38E-08	0.82	0.85	1.39E-03	0.63
LysoPC(20:3(5Z,8Z,11Z))	0.5	5.76E-11	0.87	1.01	8.67E-01	0.5
N-Acetyl-D-glucosamine 6-phosphate	8.58	7.55E-10	0.93	-	-	-

^*^AUC calculated by online tool – ROCCET (http://www.roccet.ca).

^†^*p.value* calculated by two-tailed T-test.

^‡^Fold change.

**Table 4:** AUC results of urine training and validation datasets.

Name	Urine training datasets			Urine validation datasets		
	FC(CHD/control)‡	*p.value*†	AUC∗	FC(CHD/control)‡	*p.value*†	AUC∗
N-Acetyl-D-glucosamine 6-phosphate	165.99	4.44E-09	0.96	36.91	8.29E-16	0.88
Mannitol	8.45	2.11E-11	0.92	2.62	3.99E-08	0.81
Creatine	0.41	2.85E-02	0.66	0.94	7.59E-01	0.57
Phytosphingosine	0.39	1.55E-05	0.78	1.05	2.31E-01	0.55

^*^AUC calculated by online tool – ROCCET (http://www.roccet.ca).

^†^p.value calculated by two-tailed T-test.

^‡^Fold change.

**Table 5:** Spearman correlation analysis of 512 species and identified biomarkers.

Species	Species-p.value*	Species-*q.value*†	Species-enrich	Metabolites	Coefficient	*p.value*‡	*q.value*†
*Clostridium sp. HGF2*	9.86E-05	8.65E-03	1	N-Acetyl-D-glucosamine-6-phosphate(plasma)	0.31	1.31E-03	3.57E-02
*Clostridium sp. HGF2*	9.86E-05	8.65E-03	1	N-Acetyl-D-glucosamine-6-phosphate(urine)	0.27	6.24E-03	4.99E-02
*Clostridium sp. HGF2*	9.86E-05	8.65E-03	1	Mannitol(urine)	0.26	8.52E-03	4.68E-02
*Streptococcus sp. M334*	3.13E-02	2.39E-01	1	Mannitol(urine)	0.27	5.94E-03	3.95E-02
*Streptococcus sp. M143*	3.49E-02	2.40E-01	1	Mannitol(urine)	0.2	4.26E-02	4.72E-02

^*^Species-p.value calculated by the two-tailed Wilcoxon rank-sum tests.

^†^Species-q.value and q.value calculated by false discovery rate correction.

^‡^p.value calculated by spearman correlation analysis.
