# Supplementary material for: Integrated metabolomics and metagenomics analysis of plasma and urine identified microbial metabolites associated with coronary heart disease
Source: Sci Rep. 2016 Mar 2;6:22525. doi: 10.1038/srep22525 (PMC4773756; doi:10.1038/srep22525)
Supplement: Supplementary Information [file srep22525-s1.pdf]

Integrated metabolomics and metagenomics analysis of plasma and urine identified microbial metabolites associated with coronary heart disease

Qiang Feng[1,2,3†] , Zhipeng Liu[1,4†],Shilong Zhong[5,6†], Ruijun Li[7], Huihua Xia[1,8], Zhuye Jie[1,8], Bo Wen[1], Xiaomin Chen[1], Wei Yan[7], Yanqun Fan[1], Zhenyu Guo[1], Nan Meng[1,8], Jiyan Chen[5], Xiyong Yu[5,6], Zhiwei Zhang[6], Karsten Kristiansen[1,2], Jun Wang[1,2,9,10,11] , Xun Xu[1]*, Kunlun He[7]*, Guanglei Li[1]*

1 BGI-Shenzhen, Shenzhen, 518083, China
2 Department of Biology, University of Copenhagen, Ole Maaløes Vej 5, 2200 Copenhagen, Denmark.
3 Shenzhen Engineering Laboratory of Detection and Intervention of human intestinal microbiome
4 College of Life Sciences, University of Chinese Academy of Sciences, 19A Yuquan Road, Shijingshan District, Beijing, 100094, China
5 Medical Research Center of Guangdong General Hospital, Guangzhou, Guangdong, China;
6 Guangdong Cardiovascular Institute, Guangdong Academy of Medical Sciences, Guangzhou, Guangdong, China
7 Department of cardiology, Chinese PLA general hospital, Fuxing Road 28, Beijing, 100853, China
8 Shenzhen Key Laboratory of Human commensal microorganisms and Health Research
9 Princess Al Jawhara Center of Excellence in the Research of Hereditary Disorders, King Abdulaziz University, Jeddah 21589, Saudi Arabia.
10 Macau University of Science and Technology, Avenida Wai long, Taipa, Macau 999078, China.
11 Department of Medicine, University of Hong Kong, Hong Kong.
* To whom correspondence may be addressed
† These authors contributed equally to this work.
Name and complete address for correspondence:
Dr. Guaglei Li, Fax number: +86 755 3635 2002; Tel: +86 755 3635 2017;
E-mail: liguanglei@genomics.cn
Address: Building No.11, Beishan Industrial Zone, Yantian District, Shenzhen 518083, China

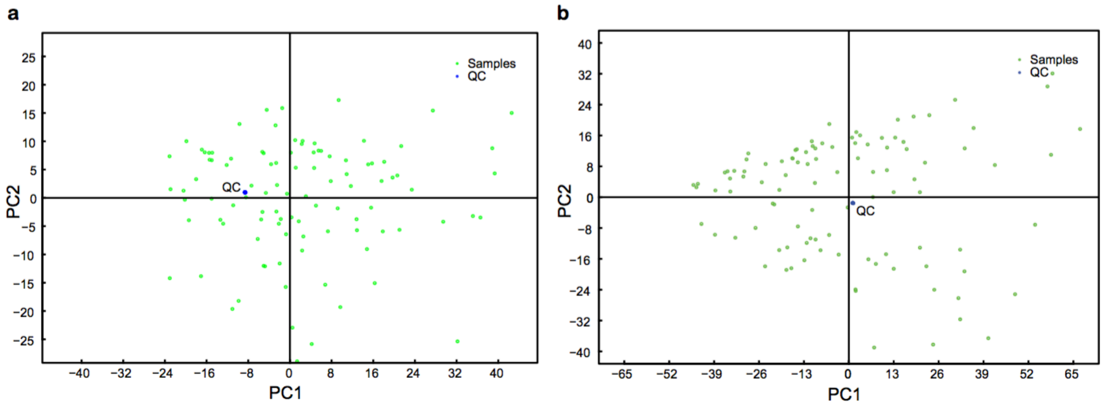

Supplementary Figure S1. QC samples PCA analysis. (**a**) PCA scores plot representation for plasma QC samples (Blue dots represent QC samples; Green dots represent other samples from CHD patients and control healthy groups). (**b**) PCA scores plot representation for urine QC samples. Both QC samples in plasma and urine clustered together demonstrating good stability and reproducibility in our current metabolomics data set**.**

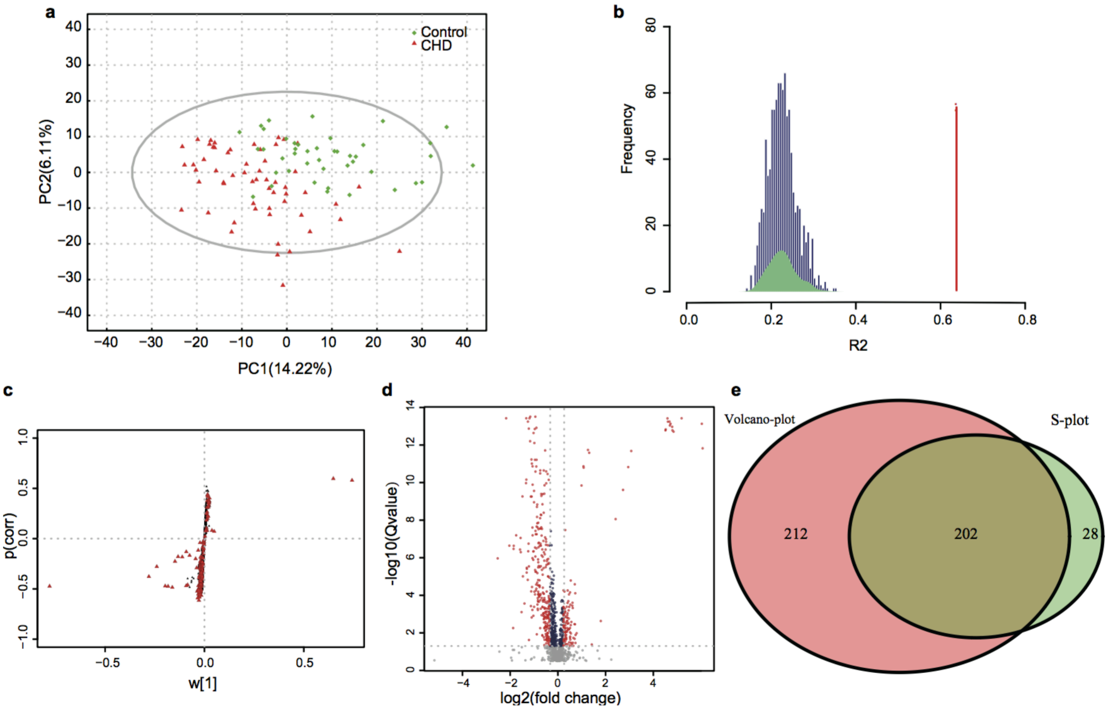

Supplementary Figure S2. Plasma metabolomics statistical analysis. (**a**) Scores plot of the PCA model distinguishing CHD patients' plasma samples from healthy controls' plasma samples. (**b**) The *R2* distribution plot of the permutation test for the PLS-DA model among plasma samples shows that the probability of the presented model random occurring is <0.001 because the model's *R2* value (red color line) is significantly distant from the randomly classified permutation distribution (n= 1000, purple color). (**c**) In S-plot, a total of 230 peaks were significantly changed in CHD patients compared with healthy controls. Red triangles indicated good correlation between variables and the model with *VIP* value greater than 1 and larger deviation, while black triangles not. (**d**) In Volcano-plot, a total of 414 peaks were significantly changed in CHD patients. Red dots showed peaks with $q.value < 0.05$ and $fold\ change > 1.2$ or $fold\ change < 0.8$, blue dots showed peaks with $q.value < 0.05$ and $0.8 \leq fold\ change \leq 1.2$, other grey dots were peaks with $q.value \geq 0.05$. (**e**) In Veen-plot, Volcano-plot and S-plot were integrated and there were 202 overlapped peaks that were significantly different between CHD patients' plasma samples and healthy controls' plasma samples.

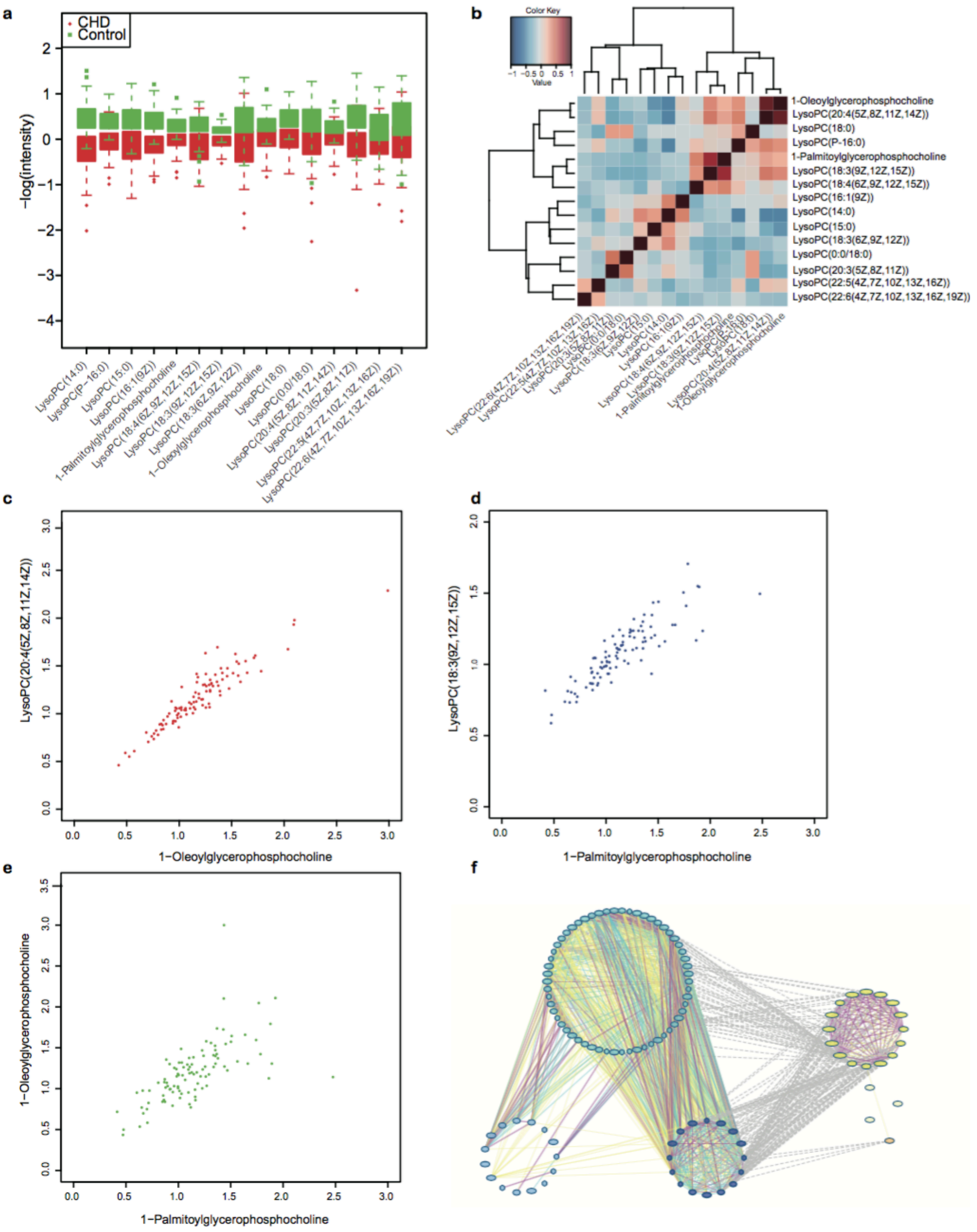

Supplementary Figure S3. Statistical analysis of 15 choline metabolites and spearman correlation analysis of 109 plasma significant annotated metabolites among CHD samples and control subjects. (**a**) Boxplot of 15 choline metabolites. (**b**) Spearman correlation analysis of 15 choline metabolites. (**c**) Scatter plot of 1-Oleoylglycerophosphocholine with LysoPC(20:4(5Z,8Z,11Z,14Z)) (*rho = 0.929, q.value = 0*) showing good and reliable positive correlation. (**d**) Scatter plot of 1-Palmitoylglycerophosphocholine with LysoPC(18:3(9Z,12Z,15Z)) (*rho = 0.874, q.value = 0*) suggesting good and reliable positive correlation. (**e**) Scatter plot of 1-Oleoylglycerophosphocholine with 1-Palmitoylglycerophosphocholine (*rho = 0.748, q.value = 0*) demonstrating good and reliable correlation. (**f**) Spearman correlation analysis of 109 annotated metabolites significantly changed in plasma demonstrated that metabolites increased in CHD patients showed relatively stronger correlations among these enriched metabolites than correlation among those decreased metabolites in CHD patients. Yellow nodes represent metabolites with increased intensities in CHD patients, blue nodes represent metabolites with decreased intensities in CHD patients. The darkness of color and sizes of nodes are correlated with significance level and fold change respectively. The deeper the color is, the smaller the *q.value* is; the larger the node size is, the more enriched metabolite in CHD patients' or healthy controls' plasma samples is. Pink lines : *rho* ≥ *0.7*, Cyan lines : *0.7 > rho* ≥ *0.6*, Yellow lines : *0.6 > rho* ≥ *0.5*, Grey lines : *rho* ≤*-0.5*.

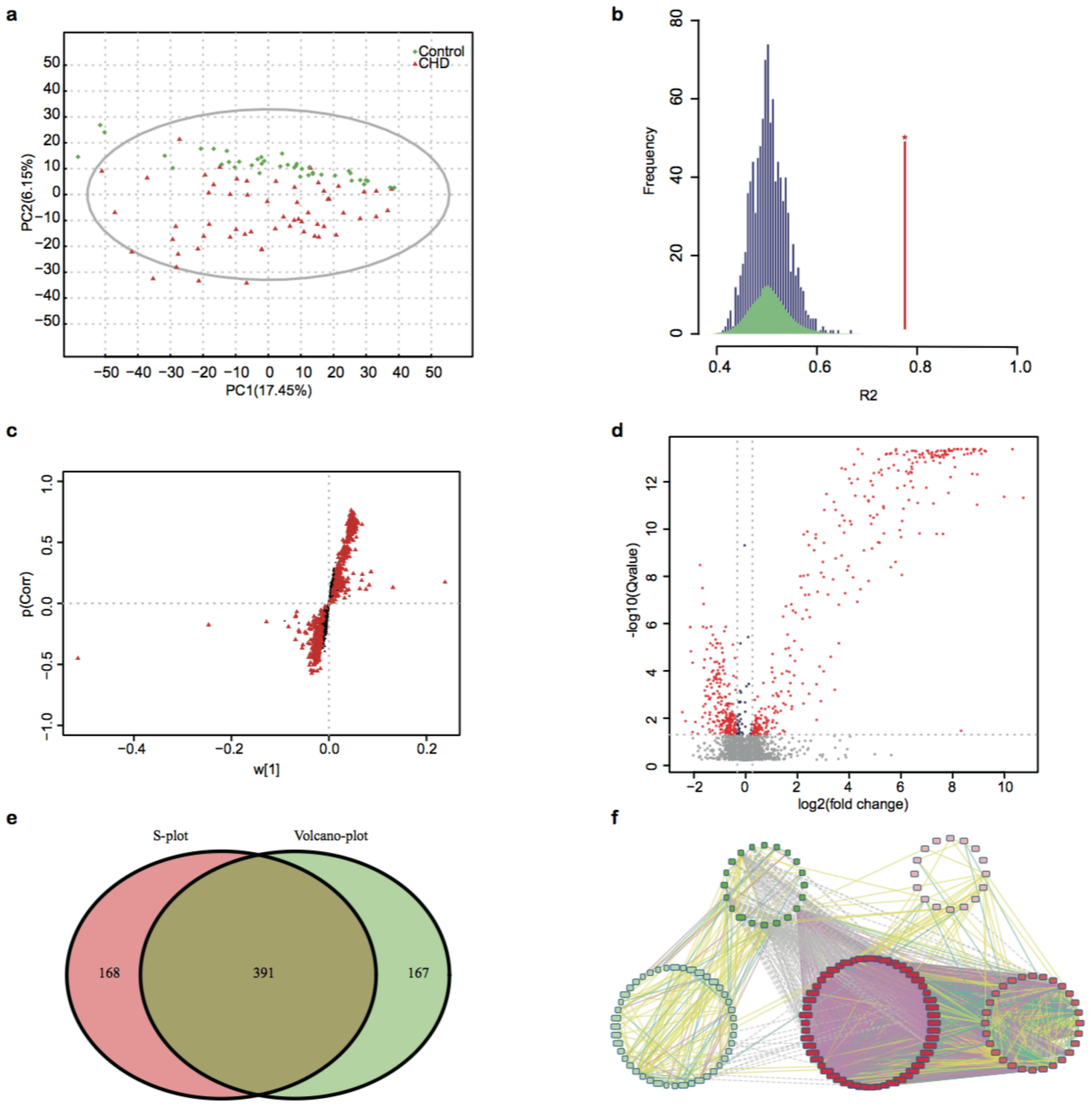

Supplementary Figure S4. Urine metabolomics statistical analysis. (**a**) Scores plot of the PCA model distinguishing CHD patients' urine samples from healthy controls' samples. (**b**) The *R2* distribution plot of the permutation test for the PLS-DA model among urine samples shows that the probability that the presented model is randomly occurring is less than 0.001 because the chosen model's *R2* value (red color) is significantly distant from the randomly classified permutation distribution (n= 1000, blue color). (**c**) In S-plot, a total of 559 peaks were significantly changed. Red triangles indicated good correlation between variables and the model with *VIP* value greater than 1 and larger deviation, while black triangles have *VIP* value less than 1. (**d**) In Volcano-plot, a total of 558 peaks were significantly changed. Red dots represent metabolites with fold change > 1.2 or *fold change* < 0.8; blue dots represent metabolites with *q.value* < 0.05 and 0.8 ≤ *fold change* ≤ 1.2; while other grey dots were peaks with *q.value* larger than 0.05. (**e**) In Veen-plot, the overlapped 391 peaks were significantly different metabolites between CHD plasma samples and control plasma samples by the integration of Volcano-plot and S-plot analysis. (**f**) Spearman correlation analysis of 160 urine significant annotated metabolites also demonstrated that metabolites increased in CHD patients showed relatively stronger correlations among these enriched metabolites than correlation among those decreased metabolites in CHD patients. .Compared with plasma significant metabolites, correlations among urine significant metabolites with smaller *q.value* either in CHD enriched metabolites or in control enriched metabolites showed relatively stronger correlations and had relatively larger coeffcients. Red nodes were metabolites with increased intensities in CHD patients, green nodes were decreased metabolites with decreased intensities in CHD patients .The deeper the color is, the more significant it is and the larger node size is, the more enriched metabolite in CHD patients' or healthy controls' plasma samples is. Red lines : *rho* ≥ *0.7*, Cyan lines : *0.7* > *rho* ≥ *0.6*, Yellow lines : *0.6* > *rho* ≥ *0.5*, Grey lines : *rho* ≤ *-0.5.*

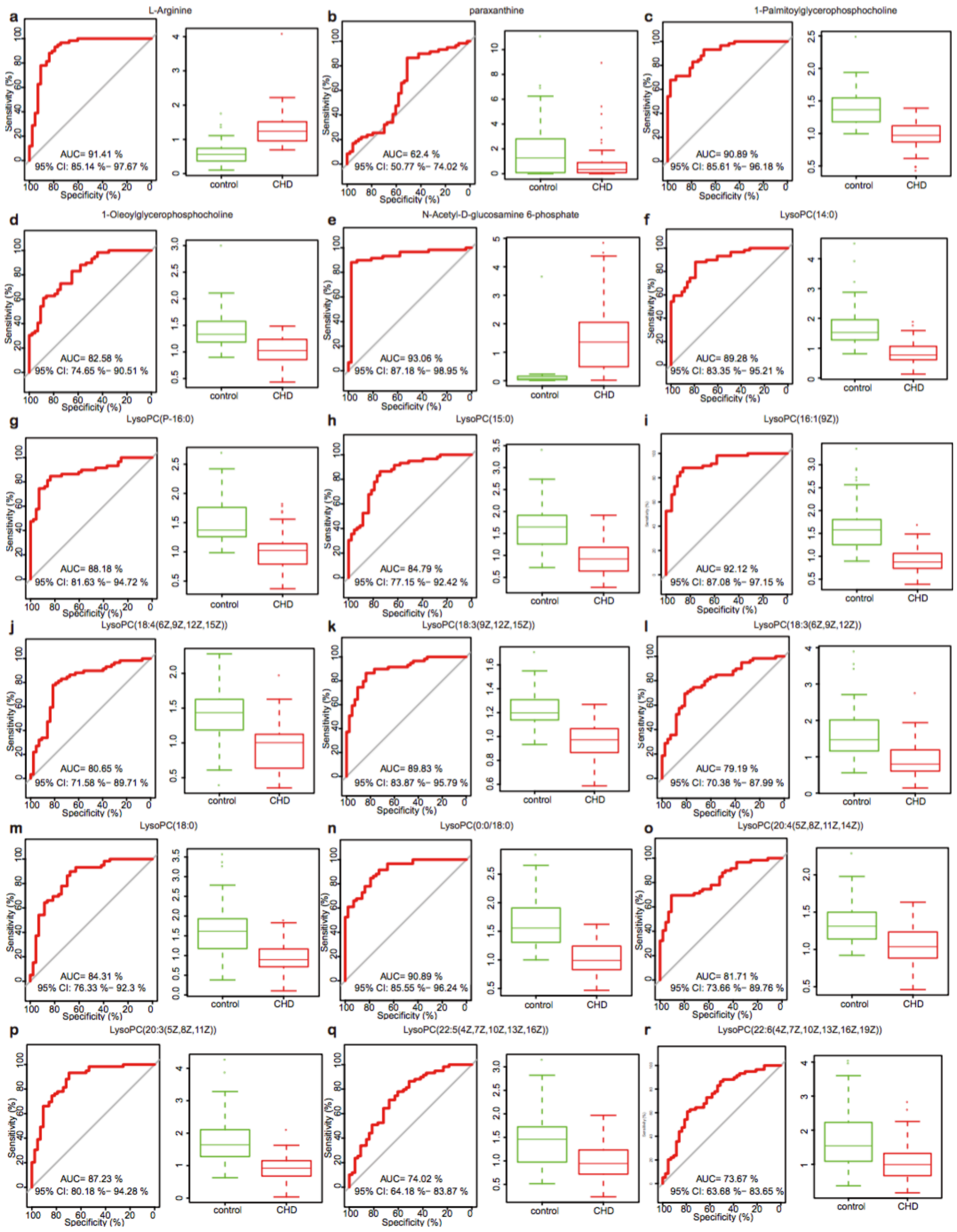

Supplementary Figure S5. Receiver operating characteristic (ROC) analysis and boxplots of 18 identified plasma potential biomarkers among 102 plasma training datasets (43 controls vs 59 CHD patients). Almost all potential biomarkers showed relatively strong discriminant capability except paraxanthine which has a AUC of 0.62.

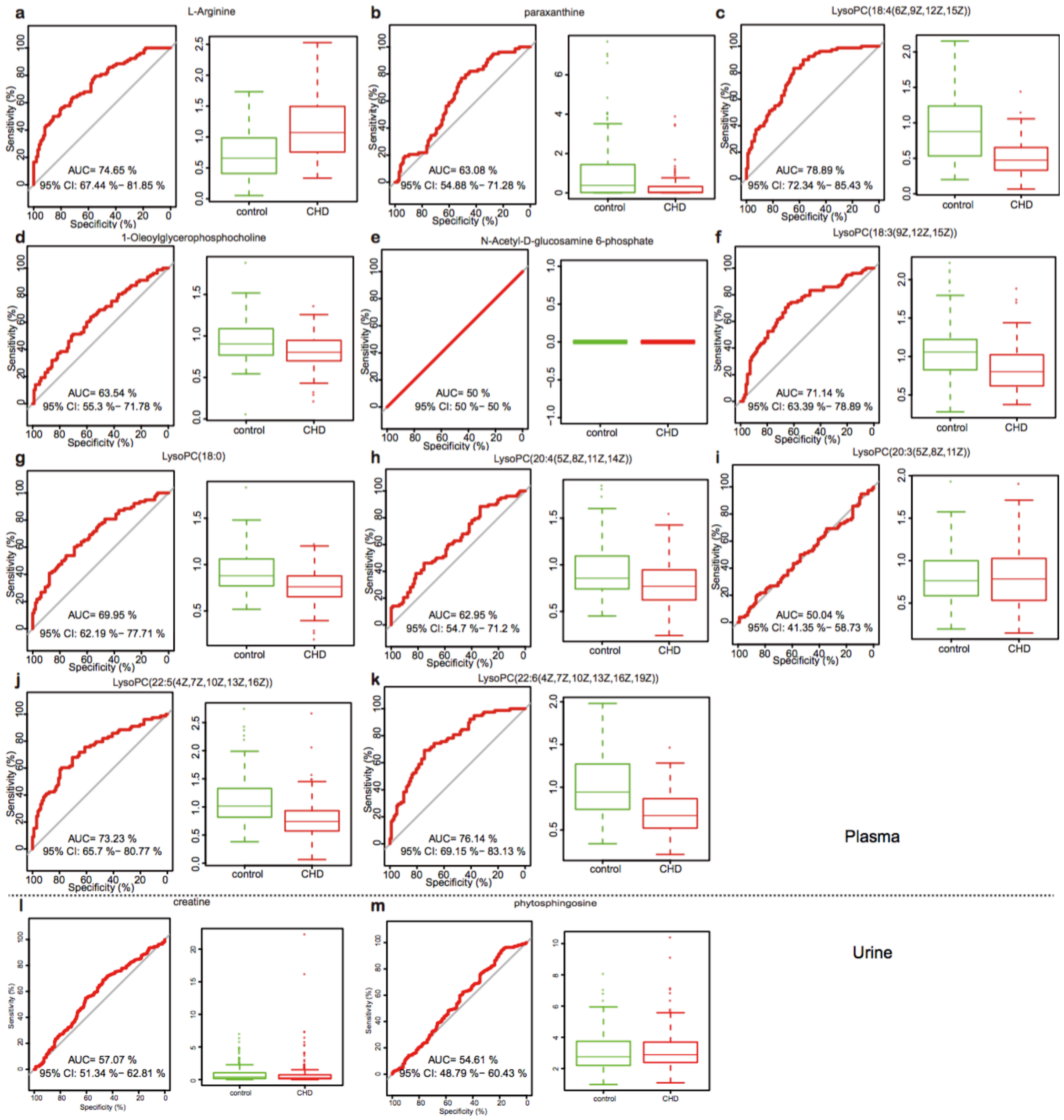

Supplementary Figure S6. Receiver operating characteristic (ROC) analysis and boxplots of 11 identified plasma potential biomarkers among 176 plasma validation datasets (98 controls vs 78 CHD patients) and 2 identified urine potential biomarkers among 395 urine validation datasets (173 controls vs 222 CHD patients). All these potential biomarkers showed diagnostic capability with AUC less than 0.80.

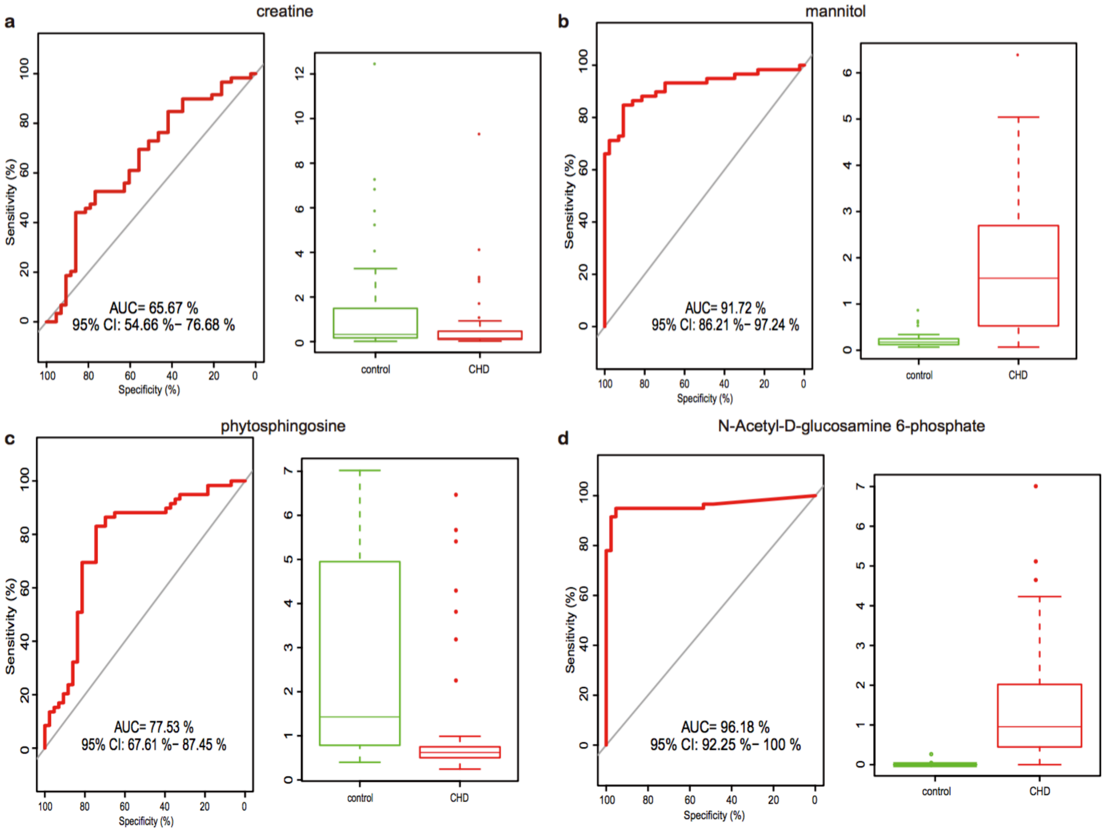

Supplementary Figure S7. Receiver operating characteristic (ROC) analysis and boxplots of 4 identified urine potential biomarkers among 102 urine training datasets (43 controls vs 59 CHD patients). Mannitol and GlcNAc-6-P showed stronger discriminate capability with AUC at 0.92, 0.96 compared with creatine and phytosphingosine with AUC at 0.66, 0.78, respectively.

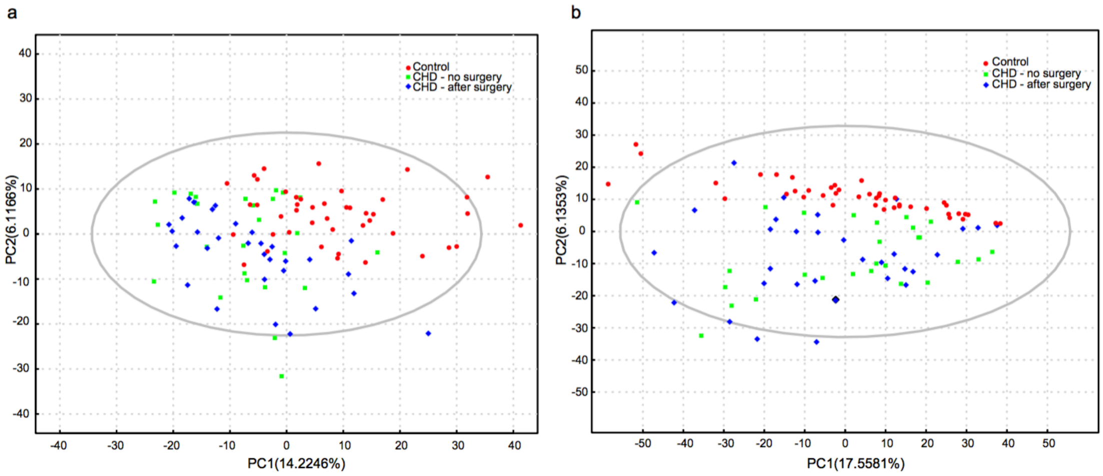

Supplementary Figure S8. PCA Scores plot of 32 postoperative patients and 27 no operative patients with 43 healthy controls. (**a**) The plasma samples of postoperative CHD patients and no operative CHD patients gathered together showing no significant difference among them. (**b**) The scattered distribution of urine samples in postoperative CHD patients and no operative CHD patients also suggested no significant difference among them.

Supplementary Table S1. The characteristics of CHD samples and control samples in the study

Characteristics	CHD patients	Control subjects	*p.value**	permuted *p.value*†
Sample number	59	43	-	-
Age (median, range), year	61, 35-79	59, 50-70	0.07	0.12
CKMB (median, range), U/L	7.40, 0.50-32.60	7.13, 2.30-14.70	0.15	0.21
ALB (median, range), g/L	37.90, 26.30-47.20	40.68, 35.40-44.10	4.06E-05‡	8.40E-03‡
ALT (median, range), U/L	26, 7-86	24.47, 10.00-56.00	0.02‡	0.02‡
TP (median, range), g/L	64.80, 55.60-75.10	73.52, 66.70-82.10	1.10E-14‡	1.00E-04‡
AST (median, range), U/L	24.30, 14.95-77.50	26.48, 19.00-45.00	0.17	0.18
CREA (median, range), µmol/L	84.20, 47.00-288.00	80.19, 50.00-123.00	0.09	0.46
HBDH (median, range), U/L	132, 91-544	151.48, 94.00-206.00	0.47	0.48
TRIG (median, range), mmol/L	1.57, 0.67-4.83	1.79, 0.37-5.09	0.96	0.18
LDLC (median, range), mmol/L	2.83, 0.87-4.99	3.32, 1.79-5.64	0.01‡	0.01‡
CHOL (median, range), mmol/L	4.48, 2.42-7.26	5.46, 3.08-8.26	4.69E-05‡	1.00E-04‡
HDLC (median, range), mmol/L	1.01, 0.66-1.59	1.32, 0.90-2.21	1.25E-07‡	1.00E-04‡
APOB (median, range), g/L	0.81, 0.52-1.21	0.92, 0.59-1.38	1.17E-03‡	5.00E-04‡
APOA (median, range), g/L	1.06, 0.56-1.90	1.30, 0.83-2.33	0.01‡	8.10E-03‡
LPA (median, range), mg/L	123.24, 23.93-1386.11	240.68, 18.00-678.00	0.92	0.48

CKMB, creatine kinase MB; ALB, albumin; ALT, Alanine aminotransferase; TP, Total Protein; AST, Aspartate transaminase; CREA, creatinine; HBDH, hydroxybutyrate dehydrogenase; TRIG, triglyceride; LDLC, low-density lipoprotein; CHOL, cholesterol; HDLC, high-density lipoprotein; APOB, apolipoprotein (b); APOA, apolipoprotein (a); LPA, lipoprotein (a). **p.value* calculated by two-tailed Student's t-test. †Permuted *p.value* calculated by PERMANOVA analysis (permutation=1000), permuted *p.value* less than 0.05 indicated significant impacts on metabolic profiles. ‡Significant clinical biochemical indicators affected metabolic profiles of CHD patients (*p.value* and permutated *p.value* less than 0.05)

Supplementary Table S2. Seven common potential biomarkers in plasma and urine samples

Category	m/z	RT(min)*	FC(CHD/ control)†	Adjusted *p.value*‡	VIP§	NAME	PATHWAYS
Potential biomarkers in plasma	185.04	10.24	0.33	3.69E-07	1.57	unknown	unknown
	311.05	10.46	29.38	1.94E-13	2.34	unknown	unknown
	310.04	10.46	28.41	1.65E-13	2.35	unknown	unknown
	309.05	10.46	26.40	9.08E-14	2.35	unknown	unknown
	202.04	9.72	0.38	2.70E-04	1.41	unknown	unknown
	324.04	9.33	8.58	2.08E-12	2.16	N-Acetyl-D-glucosamine 6-phosphate‖¶	Amino sugar and nucleotide sugar metabolism
	125.01	10.46	7.86	1.50E-11	2.31	unknown	unknown
Potential biomarkers in urine	185.04	10.30	0.34	1.70E-03	1.62	unknown	unknown
	311.05	10.51	59.41	3.75E-14	2.26	unknown	unknown
	310.04	11.25	110.51	5.70E-14	2.47	unknown	unknown
	309.05	11.28	12.65	1.28E-07	2.03	unknown	unknown
	202.04	9.77	0.58	8.10E-04	1.06	unknown	unknown
	324.04	10.25	165.99	4.28E-14	2.30	N-Acetyl-D-glucosamine 6-phosphate‖¶	Amino sugar and nucleotide sugar metabolism
	125.01	11.18	158.93	5.11E-14	2.42	unknown	unknown

*Retention time. †Fold change. ‡Adjusted *p.value* calculated by the two-tailed Wilcoxon rank-sum tests after false discovery rate correction. §VIP (Variable Importance for Projection), one indicator reflecting the capability of the variables to explain Y. ‖Metabolites matched with commercial available reference standards. ¶Metabolites matched characteristic peaks but mismatching retention time with commercial available reference standards.

Supplementary Table S3. Spearman correlation analysis of the 7 common metabolites

Plasma	Urine	Coefficient	*p.value**	Adjusted *p.value* †	Enrichment
311.05	311.05	0.812	3.18E-25	3.12E-24	CHD
310.04	310.04	0.803	3.00E-24	1.63E-23	CHD
324.04	324.04	0.747	1.83E-19	5.60E-19	CHD
125.01	125.01	0.687	1.38E-15	3.22E-15	CHD
202.04	202.04	0.642	0	0	Control
309.05	309.05	0.556	1.96E-09	4.18E-09	CHD
185.04	185.04	0.547	4.48E-09	8.78E-09	Control

**p.value* calculated by spearman correlation analysis. †Adjusted *p.value* calculated by false discovery rate correction.

Supplementary Table S4. Spearman correlation analysis of clinical data and identified biomarkers

**Potential Biomarkers**	**Clinical biochemical indicators**	**Coefficient**	***p.vlaue****	**Adjusted *p.value* †**
LysoPC(18:0)	CHOL	0.52	2.39E-08	7.89E-07
LysoPC(P-16:0)	HDLC	0.56	8.45E-10	7.39E-08
LysoPC(18:0)	HDLC	0.55	2.56E-09	1.29E-07
LysoPC(14:0)	TP	0.59	5.19E-11	1.71E-08
LysoPC(18:0)	TP	0.57	3.13E-10	5.16E-08
LysoPC(16:1(9Z))	TP	0.56	9.08E-10	7.39E-08
LysoPC(15:0)	TP	0.56	1.12E-09	7.39E-08
1-Palmitoylglycerophosphocholine	TP	0.55	2.74E-09	1.29E-07
LysoPC(18:3(9Z,12Z,15Z))	TP	0.54	6.40E-09	2.64E-07
LysoPC(20:3(5Z,8Z,11Z))	TP	0.53	8.31E-09	3.05E-07
LysoPC(18:4(6Z,9Z,12Z,15Z))	TP	0.51	3.63E-08	1.09E-06

*****_p.value_ calculated by spearman correlation analysis. †Adjusted *p.value* calculated by false discovery rate correction.

Supplementary Table S5. Spearman correlation analysis of 65 KOs and identified biomarkers

KO	KO-*p.value**	KO-enrich	metabolites	Coefficient	*p.value*†	*q.value*‡
K01443	1.87E-02	1	N-Acetyl-D-glucosamine-6-phosphate(plasma)	0.28	4.16E-03	1.56E-02
K01788	5.48E-04	1	N-Acetyl-D-glucosamine-6-phosphate(plasma)	0.39	5.55E-05	2.71E-03
K01443	1.87E-02	1	N-Acetyl-D-glucosamine-6-phosphate(urine)	0.26	7.17E-03	2.15E-02
K01788	5.48E-04	1	N-Acetyl-D-glucosamine-6-phosphate(urine)	0.31	1.40E-03	8.56E-03
K02755	1.04E-03	1	Mannitol	0.36	2.63E-04	5.11E-03
K02756	1.78E-03	1	Mannitol	0.33	6.31E-04	6.20E-03
K02759	1.52E-03	1	Mannitol	0.33	8.64E-04	6.79E-03
K02760	7.79E-05	1	Mannitol	0.33	8.48E-04	6.79E-03
K02768	7.00E-03	1	Mannitol	0.31	1.73E-03	9.25E-03
K02769	1.53E-02	1	Mannitol	0.27	6.99E-03	2.12E-02
K02777	4.36E-04	1	Mannitol	0.32	1.09E-03	7.10E-03
K02793	3.13E-03	1	Mannitol	0.30	2.33E-03	1.06E-02
K02794	1.87E-02	1	Mannitol	0.29	3.61E-03	1.41E-02
K02808	1.56E-02	1	Mannitol	0.24	1.52E-02	3.71E-02
K02809	1.91E-03	1	Mannitol	0.27	6.17E-03	2.00E-02
K02818	9.43E-04	1	Mannitol	0.31	1.79E-03	9.25E-03
K02821	6.53E-04	1	Mannitol	0.32	9.40E-04	6.79E-03
K11202	3.49E-03	1	Mannitol	0.30	2.11E-03	9.78E-03

*KO-*p.value* calculated by the two-tailed Wilcoxon rank-sum tests. †*p.value* calculated by spearman correlation analysis.
‡*Q.value* calculated by false discovery rate correction.

Supplementary Materials and Methods

Experimental Materials

Formic acid and methanol (HPLC grade) were purchased from Fisher Scientific Corporation (Loughborough, UK). Water used in the experiments was obtained from a Milli-Q Ultra-pure water system (Millipore, Billerica, MA). An Agilent ZORBAX ODS C-18 column (150 mm×2.1 mm, 3.5 μm, Agilent, USA) was used for all analysis.

Clinical samples collections

Venous blood and midstream urine were collected in the morning before breakfast from all participants. Venous blood collected by Vacuette EDTA blood collection tubes were centrifuged at 2200xg for 5 min at 4 °C to obtain plasma samples. The plasma and urine samples were stored at −80 °C until use.

Fecal samples were mechanically homogenized with a sterile spatula, and then aliquots containing 1g of stool in a 12ml sterile cryovial were made using the Sarstedt stool sampling system (Sarstedt, Nümbrecht, Germany). The aliquots were then stored in freezers at -20 °C and transported to the laboratory with ice pack within 48 h after collection. After that, fecal samples were stored at −80 °C until use.

Samples preparations for HPLC-MS experiments

To extract the low-molecular-weight metabolites (<1500Da) in the plasma and urine samples, some modifications were made to the procedures reported previously[1]. Before experiment, all plasma and urine samples were thawed on ice and a "quality control" (QC) sample was made by mixing and blending equal volumes (10μL) from each plasma or urine samples which was applied to estimate a "mean" profile representing all the analytes encountered during analysis. Then low molecular weight metabolites (<1500Da) were extracted from the plasma and urine samples using the following procedure: plasma samples were mixed with methanol (1:2 v/v) while urine samples were mixed with methanol (1:1 v/v) to precipitate protein. The plasma and urine sample mixture were centrifuged at 14000x g for 10 min at 4°C and then supernatant was transferred into a 1.5 mL polypropylene tube for metabolic profiling by HPLC−MS.

HPLC-MS experiments

Shimadzu Prominence HPLC system (Shimadzu) was coupled to a LTQ Orbitrap Velos instrument (Thermo Fisher Scientific, MA, USA) set at 30000 resolution to acquire HPLC-MS data. An Agilent ZORBAX ODS C18 column (150 mm×2.1 mm, 3.5 μm, Agilent, USA) was used. Sample analysis was

performed in positive ion modes with a spray voltage of 4.5 kV and capillary temperature of 350°C. The mass scanning range was 50−1500 m/z. The flow rates of nitrogen sheath gas and nitrogen auxiliary gas were set to 30 L/min and 10 L/min, respectively. The HPLC−MS system was run in binary gradient mode. Solvent A was 0.1% (v/v) formic acid/water, and solvent B was 0.1% (v/v) formic acid/methanol. The gradient was as follows: 5% B at 0 min, 5% B at 5 min, 100% B at 8 min, 100% B at 9 min, 5% B at 18 min, and 5% B at 20 min. The flow rate was 0.2 mL/min. To ensure system equilibrium, the pooled QC sample was injected five times at the beginning. QC sample was injected every five samples during samples detection to further monitor the system stability.

DNA extraction from fecal samples

Fecal samples were thawed on ice and DNA was extracted using the Qiagen QIAamp DNA Stool Mini Kit (Qiagen) according to manufacturer`s instructions. Extracts were treated with DNase-free RNase to eliminate RNA contamination. And DNA quantity was measured using NanoDrop spectrophotometer, Qubit Fluorometer (with the Quant-iTTMdsDNA BR Assay Kit) and gel electrophoresis.

DNA library construction and metagenomic sequencing of fecal samples

DNA library construction was performed following the manufacturer's instruction (Illumina). The detailed method was described previously. Basically, the following procedure was performed: cluster generation, template hybridization, isothermal amplification, linearization, blocking and denaturation, and hybridization of the sequencing primers. One paired-end (PE) library was constructed with insert size of 350 bp for each sample, followed by a high-throughput sequencing to obtain around 30 million PE reads with a of length 2x100bp. High-quality reads were obtained by filtering low-quality reads with ambiguous 'N' bases, adapter contamination and human DNA contamination from the Illumina raw reads, and by trimming low-quality terminal bases of reads simultaneously.

References

1. Luan, H. et al. Serum metabolomics reveals lipid metabolism variation between coronary artery disease and congestive heart failure: a pilot study. Biomarkers **18**, 314−321 (2013).
